# Uncoding the interdependency of tumor microenvironment and macrophage polarization: insights from a continuous network approach

**DOI:** 10.3389/fimmu.2023.1150890

**Published:** 2023-05-22

**Authors:** Ugo Avila-Ponce de León, Aarón Vázquez-Jiménez, Pablo Padilla-Longoria, Osbaldo Resendis-Antonio

**Affiliations:** ^1^ Programa de Doctorado en Ciencias Biológicas, Universidad Nacional Autónoma de Mexico, Ciudad de Mexico, Mexico; ^2^ Human Systems Biology Laboratory, Instituto Nacional de Medicina Genómica (INMEGEN), Ciudad de Mexico, Mexico; ^3^ Institute for Applied Mathematics (IIMAS), Universidad Nacional Autónoma de Mexico, Ciudad de Mexico, Mexico; ^4^ Coordinación de la Investigación Científica - Red de Apoyo a la Investigación, Universidad Nacional Autónoma de México (UNAM), Ciudad de Mexico, Mexico; ^5^ Centro de Ciencias de la Complejidad (C3), Universidad Nacional Autónoma de Mexico, Ciudad de Mexico, Mexico

**Keywords:** macrophage polarization, fuzzy logic, ordinary differential equations, systems immunology, gene regulatory network, cancer immunology

## Abstract

The balance between pro- and anti-inflammatory immune system responses is crucial to preventing complex diseases like cancer. Macrophages are essential immune cells that contribute to this balance constrained by the local signaling profile of the tumor microenvironment. To understand how pro- and anti-inflammatory unbalance emerges in cancer, we developed a theoretical analysis of macrophage differentiation that is derived from activated monocytes circulating in the blood. Once recruited to the site of inflammation, monocytes can be polarized based on the specific interleukins and chemokines in the microenvironment. To quantify this process, we used a previous regulatory network reconstructed by our group and transformed Boolean Network attractors of macrophage polarization to an ODE scheme, it enables us to quantify the activation of their genes in a continuous fashion. The transformation was developed using the interaction rules with a fuzzy logic approach. By implementing this approach, we analyzed different aspects that cannot be visualized in the Boolean setting. For example, this approach allows us to explore the dynamic behavior at different concentrations of cytokines and transcription factors in the microenvironment. One important aspect to assess is the evaluation of the transitions between phenotypes, some of them characterized by an abrupt or a gradual transition depending on specific concentrations of exogenous cytokines in the tumor microenvironment. For instance, IL-10 can induce a hybrid state that transits between an M2c and an M2b macrophage. Interferon- γ can induce a hybrid between M1 and M1a macrophage. We further demonstrated the plasticity of macrophages based on a combination of cytokines and the existence of hybrid phenotypes or partial polarization. This mathematical model allows us to unravel the patterns of macrophage differentiation based on the competition of expression of transcriptional factors. Finally, we survey how macrophages may respond to a continuously changing immunological response in a tumor microenvironment.

## 1 Introduction

Macrophage phenotypes contain a spectrum of stages whose selection is ruled by feedback loops between transcription factors and exogenous signals from the microenvironment (1–4). Evidently, there is a close relationship between the exogenous signals that set a specific environment and the macrophage functionality. Interestingly, the signaling microenvironment drives the polarization of the macrophage to eliminate tumor cells, or it reduces the cytokine storm and removes the cells causing the permanent immune response (5, 6). In other words, different combinations of cytokines in the microenvironment can be agonists or antagonists of macrophage polarization. This fact reveals that macrophages are essential components in shaping the inflammatory response from the initial to the resolution phase (7–10). Despite this specific control on the macrophage state having been reported, its regulatory mechanisms are not well understood yet (11). In this context, two immediate questions emerge in cancer studies. The first is how the tumor microenvironment modulates the polarization of macrophages for thriving cancer cells and developing the aggressive phenotype. The second is how to change the signal microenvironment to design and improve therapeutical applications.

In a glimpse, the whole spectrum of macrophage phenotypes starts with the monocytes (M0), which circulate in the blood and are in charge of sensing the released chemokines at the site of inflammation (12). Once in the microenvironment, macrophages M0 polarize to a steady state in response to different signals. In general, macrophages have anti-inflammatory and pro-inflammatory phenotypes. Pro-inflammatory phenotypes are related to M1-type macrophages, while the anti-inflammatory phenotypes are associated with regulatory/wound healing behavior denoted as M2a, M2b, M2c, and M2d macrophages. Interestingly, the signals produced by macrophages reinforce their phenotype and, in turn, reshape the environment. M1 macrophages express NFκB or STAT1 and secrete TNF- α and IL-12, which correlate with a microbicidal and pro-inflammatory response. The M2 anti-inflammatory phenotype has several sub-phenotypes (M2a, M2b, M2c, and M2d) with a higher range of complex responses than the M1 phenotype (13). Hence, M2a macrophages express STAT6 and secrete IL-10, TGFB, and IL-1RA, whose activity is associated with fungal and helminth infections, inhibition of Th1, and a Th2 response (14). M2b macrophages express ERK and AP-1, and secrete IL-10, TNF- α, and IL-1, while it prevails in immune regulation (15). M2c macrophages express STAT3 and secrete IL-10 and TGFB, which are involved in tissue repair, matrix remodeling, and immunosuppressive behavior (16). M2d macrophages express HIF1-α or a defective NFκB, enhance STAT1, and induce the secretion of IL-10, TGFB, and VEGF. Interestingly, there are shreds of evidence that the M2d phenotype is associated with malignant tumor development by regulating the immune system and enhancing angiogenesis, metastasis, and tumor growth (17–19).

The tumor microenvironment can reversibly transit among the macrophage polarization states through a miscellaneous of external stimuli (20). This fact supports the idea that macrophages are highly plastic and heterogeneous in their responses and phenotypes during polarization (3, 21). Even more, there are several articles describing macrophage polarization through a spectrum model of additional phenotypes, further than the well-established M1 and M2 phenotypes, constrained by the signaling and metabolites into the environment. In particular, the authors in (22) suggested a regulatory network that reveals novel TFs that coordinate to respond to inflammation as a function of the signaling environment condition. Furthermore, a recent study has associated gene expression profiles with different stimuli through transcriptome and network modeling. The authors found that macrophages when exposed to stimuli associated with chronic inflammation (TNF-α and prostaglandin E2), developed different activation programs commanded by STAT4 (23). In addition, they concluded that a macrophage polarized to a phenotype does not its behavior by adding another signaling component linked to that phenotype. However, the addition of metabolic (fatty acids, glycolysis, and oxidative phosphorylation components) or chronic inflammation components into the environment favors the changes in the macrophage phenotype. Evidence of these metabolic adaptations in macrophages has been described elsewhere in more detail (24, 25). More recently, macrophage polarization has been analyzed by next-generation sequencing technology like single cell (SC). Because of the detail provided by this technology, it allows us to survey how macrophages behave in response to an immune threat or stimuli they encounter (26). An interesting observation obtained from SC technology is that once a pro-inflammatory cytokine activates a macrophage, there is a small proportion of macrophages that secretes IL-10 to balance and avoid a hyper-inflammatory scenario (27). Notably, these studies point out that there is no transcriptional difference between the macrophages that secrete pro-inflammatory cytokines compared to the macrophages that secrete IL-10. This generation of macrophage that secretes IL-10 is a response based on the cell density to maintain inflammatory homeostasis (28). Another application of SC in ovarian and pancreatic ductal adenocarcinoma has demonstrated that if the balance of macrophage polarization tilts to a pro-inflammatory secretion, then the clinical output is associated with a good prognosis (27, 29). Conversely, the anti-inflammatory macrophages are a highly interactive hub with the tumor microenvironment and can generate pairs of ligand-receptor with a bad prognosis and low survival rate (29). Under this assumption, mixed cellular phenotypes or hybrid stages in macrophage polarization is a feasible situation induced by the activity of cytokines, membrane receptors, and transcription factors in the microenvironment. In agreement with this idea, hybrid macrophages have been detected experimentally (30), however, the diversity of hybrid macrophage states has not been fully described. Curiously, the same signal that polarizes macrophages from a monocyte can return it to the same state from another phenotype. For example, M1 macrophages can polarize reversibly into M2a (if IL-4 is in the microenvironment), M2c (If IL-10 is present), and M2d (If IL-6 is present), while M2c can polarize reversibly into M2a (If IL-4 is being secreted) (17–19, 31). Starting from Boolean dynamic modeling, these patterns of transitions can be simulated by reconstructing a cell fate map from the perturbations of the steady-state attractors and evaluating which phenotypes are fixed and cannot move to another state, despite a perturbation (21).

Given the plethora of massive amounts of stimuli in a tumor microenvironment, the possibility of understanding the dynamics of macrophage behavior becomes challenging. To elucidate and understand these mechanisms, there has been an intense activity to integrate signal responses and molecular mechanisms into complex regulatory networks (1, 3, 21, 32). Mathematical and computational analysis of these regulatory networks has become a useful approach to understanding the multistability underlying cell fates in macrophages. Two approaches overcome this list of methods, the Boolean and ODE formalisms. One advantage of the Boolean scheme is that it can reach different stable state configurations, called attractors, without the need for kinetic parameters (25, 26, 28). Hence, our team, through a Boolean network approach, accomplished a metabolic reconstruction and postulated that the static attractors were associated with different types of macrophage phenotypes (comprising M0, M1, M2, and hybrid stated among them) (21). Despite its utility, the Boolean approach does not capture fine-grain details of the continuous nature of the gene expression profiles, which in most cases is an important variable to explore continuous phenotypes. With the purpose to overcome this situation, some mathematical models have been reported simulating macrophage polarization and plasticity using ordinary differential equations (ODEs) (33–36). Particularly, an important mathematical result with experimental evidence is the one developed by the authors in reference (35). The authors constructed a complete model with a total of 80 differential equations to explain the complex intracellular signaling pathways in macrophage polarization. They used three types of activation (IFNG (M1a), IL-4 (M2a), and oxidative stress (M2d)) for identifying which are the most important transcription factors driving each phenotype. In another paper, the authors developed a system of ODEs based on a mathematical transformation of the Wilson-Cowen structure equation (34). This system of ODEs was used to explain the bifurcation diagram of the M1 and the M2 based on the expression of NFκB and STAT6 respectively. As a result, they supplied evidence in favor of hybrid phenotypes. However, despite these and other scientific endeavors, none of these approaches have portrayed the landscape between macrophage hybrid phenotypes and their transition as a function of the microenvironment. With the purpose to contribute to this direction, here we applied a mathematical model of fuzzy logic onto a set of previously reported Boolean attractors obtained from a transcriptional regulatory network of macrophages (21). Mathematical models based on fuzzy logic have been applied to explore the phenotype landscape in complex systems such as regulatory networks in flower development (37, 38). As a result, the continuous model of the transcriptional regulation of macrophage polarization allowed us to: 1) evaluate the quantitative changes in the external (exogenous cytokines) and internal (transcriptional factors) components, and 2) assess their potential to cause transitions among previously reported macrophage phenotypes (21).We focused specifically on the M1 and M2 subtype transitions, due to their importance in balancing the pro- and anti-inflammatory responses in the tumor microenvironment. Through this approach, we untangled the robust circuitry underlying the M1 and M2 subtype macrophages and the shift between a cytotoxic and regulatory immune response when early differentiation of macrophages occurs. The results in this article may be useful to understand the dynamics of the mechanisms of the response of macrophages, how its adaptability and plasticity can be handled by the signals in the microenvironment. Potentially, understanding the complex relationship between macrophage phenotype and signals from microenvironments will contribute to the design of effective strategies in cancer studies, where the balance of pro- and anti-inflammatory response is moving to favor the malignant phenotype.

## 2 Material and methods

### 2.1 Network reconstruction and Boolean states

**Figure 1:**
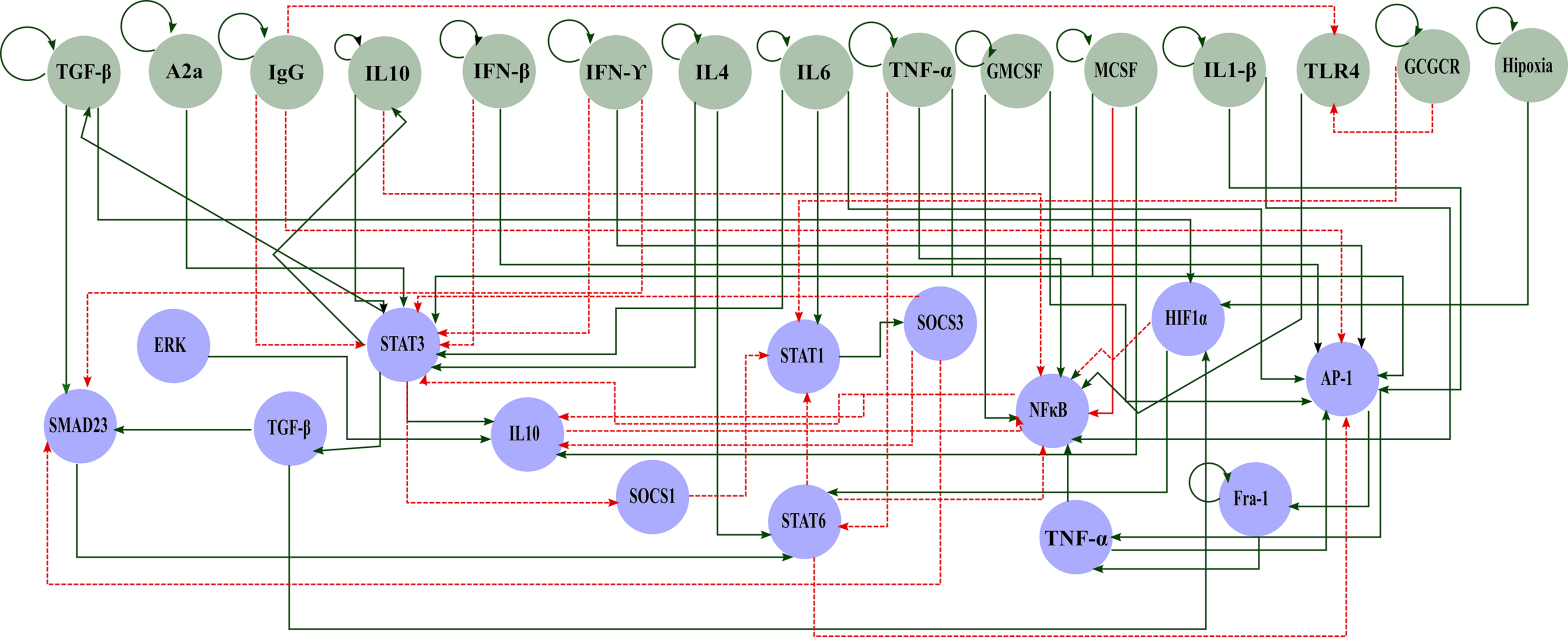
Gene regulatory network polarization in a tumor microenvironment. Green circles represent components of the extracellular space and blue circles of the internal machinery. Solid green arrows represent activation and dashed red arrows inhibition.

We start our analysis by considering a concise network of the molecular basis of macrophage polarization and the Boolean attractors obtained from it. Both sources of data were previously reported by our group of researchers in (21).In summary, our reconstructed network was obtained through a bottom-up approach, where we set up the signaling network by literature research on interactions. The resulting transcriptional regulatory network (TRN) has 29 nodes and 60 interactions and consists of two parts: the extracellular component (green nodes) and the master TFs (blue nodes) (**Figure 1**). As reported before, we simulate the dynamics of a macrophage in a tumor microenvironment by the Boolean approach. Then, Boolean attractors obtained from this network were classified into pure or mixed states among the five experimentally proven macrophage phenotypes: M1, M2a, M2b, M2c, and M2d. In global terms, we used the following criteria to label these attractors. For the M1 macrophages, we considered that the TFs associated with these phenotypes were STAT1 and NFκB (39, 40). The activation of at least one of these TFs is implicated with secreted cytokines that eliminate tumor growth and enhance a pro-inflammatory condition (41, 42). M2 activation is associated with pro-tumorigenic outcomes. The key TF implicated in M2a’s Th2 response and profibrotic is STAT6 (43)- (44, 45). STAT6, at the same time, can inactivate the functions of NFκB and STAT1 through SOCS1, avoiding the M1 phenotype. The activation of the M2b macrophage is quite complex, with the inclusion of immune complexes (like immunoglobulin G (IgG)) and interleukin-1 receptors (IL1-R) (for example IL1-β) (15, 46). The M2b macrophage has the ability to secrete not only anti-inflammatory cytokines but pro-inflammatory cytokines is M2b (47, 48). The M2c emerges from the stimulation of IL-10 [the most important cytokine with an anti-inflammatory capacity and associated with bad prognosis in cancer (49, 50)]. STAT3 is the condition for the anti-inflammatory capacity and inhibitor of the secretion of pro-inflammatory cytokines (51). Finally, the M2d macrophage is activated via a co-stimulation of adenosines and TLR4, as well as the activation of HIF1-α (52). This macrophage is implicated in processes like angiogenesis and tumor progression, which is why they are also known as tumor-associated macrophages (52, 53). The full description and characterization of the network used in this work is in (21). The list of Boolean attractors and their classification in macrophage states can be reviewed in the **Supplementary Material**.

### 2.2 The transformation from discrete to continuous using the fuzzy logic approach

The Boolean approach allowed us to explore the epigenetic landscape that emerged from a regulatory network, by considering that each element in the network can be in one of two states, 0 or 1. To overpass this limitation, in this paper we neglect this assumption and consider that the elements in the regulatory network can take continuous and normalized values of expression. Extracellular and intracellular signals may behave as continuous variables, therefore we identified additional attractors with biological relevance. The model transformation from discrete to continuous was carried out using the booleanTODE function in the BoolNetPerturb R package. In our model, we used a fuzzy logic model based on probabilistic rules and the Villareal method (29, 31).

The regulatory network consists of 29 nodes (i) interacting with each other with an expression level at a time t stated by $q_{i}\left(t\right)$ . As in the Boolean model, the state of a node is regulated by its interaction with other nodes, which can be represented by the fuzzy logic composition $\omega_{i}\left(q_{i1}\ldots q_{2q}\right)$ ω_i_ (q_i1_ … q_2q_). The characteristic function with that logic structure was parameterized through the following expression:


$$\phi \left[\omega_{i}\right]=\frac{1}{1+exp\left[-b\left(\omega_{i}-\omega_{thr}\right)\right]}$$

Where b indicates the saturation rate of the node from being unexpressed (value equal to zero) to an expressed state (value equal to one). A small value of b settles that the shift is gradual, and a large value settles a sharp shift (32). Meanwhile, $\omega_{thr}$ represents the threshold between inactivation and activation, we set the value to 0.5. $\omega_{i}$ was defined according to the interaction of their nodes in the following manner:

**Table:** 

Boolean rules	Probabilistic Rules
q AND p	q*p
q OR p	q+p−q*p
NOT p	1−p

suppose p and q are nodes in a fictional regulatory network. Once probabilistic rules are applied, we end with 29 differential equations, which represent each of the 29 nodes in the regulatory network.

### 2.3 Dynamics of our model in a continuous scale

The dynamical simulation of the normalized expression levels of each $q_{i}\left(t\right)$ was determined by the regulatory network described by a set of ODES in the form:


$$\frac{dq_{i}}{dt}=\theta \left[\omega_{i}\right]-\alpha_{i}q_{i}$$

where α _i_ denotes the decay rate of the expression of the node. We set the value of each $\alpha_{i}$ to one so that the expression level of the node at its stationary state is merely determined by the degree of the truth of the fuzzy proposition $\omega_{i}$ (32, 33). The detail of the ODE system used for simulating the macrophage regulatory network can be seen in **Supplementary Material** and https://github.com/resendislab/DifEquations-macrophage.

### 2.4 Polarization analysis

The fuzzy logic transformation allowed us to evaluate the alteration of the inputs on a continuous scale between [0,1] We modeled the polarization process of a monocyte (absence of a polarizing cytokine) to evaluate the final steady states in a specific cytokine environment. To achieve the description of the macrophage phenotypes we consider that a node is activated if its steady-state value $q_{i}\geq 0.75$ , and inactivated when the value is $q_{i}\leq 0.25$ The intermediate range $0.25<q_{i}<0.75$ may correspond to a hybrid phenotype. Evaluating all the possible initial conditions in this continuous regulatory network is conceptually impossible because we have to set infinite initial conditions for each node lying in the range [0,1] . To overpass, this limitation, we only validated specific phenotypes obtained in the discrete Boolean setup and evaluated how specific perturbations affect the steady-state of a specific phenotype. All steady states that lay in the intermediate zone will be considered to be hybrid phenotypes (phenotype coexistence). The code was adapted from (32).

### 2.5 Network reconstruction

We reconstructed a macrophage regulatory network using a literature search for interactions between external components and how this affects the activation or inhibition of transcriptional factors associated with macrophage polarization (21). The feasible space of phenotypes was obtained using a Boolean approach. Briefly, we assumed that each node in the network can be in one of two states (0,1) , and their dynamic behavior is entirely governed by a Boolean function, which is defined by the logical rules of our network (34). Our regulatory network includes 40 nodes, excluding intermediate nodes in the lineal array, for example, node a activates node b, and then node b activated node c. This interaction was reduced to only node an activating node c because eliminating node b did not affect the global interaction. The reduction resulted in a final network of 29 nodes. 14 nodes are associated with important transcription factors and 15 nodes with interleukins or metabolic byproducts present in a tumor microenvironment. Macrophage behavior depends greatly on the microenvironment being recruited so we focused on these microenvironments: pro-M1, pro-M1a, pro-M2a, pro-M2b, pro-M2c, and pro-M2d. Finally, we explored 4 breast cancer-associated microenvironments as in (21). All the implemented code for the methodology and analysis is available at https://github.com/UgoAvila/Differential-equations.

## 3 Results

### 3.1 Consequence of the exogenous tumor microenvironment on macrophage polarization

**Figure 2:**
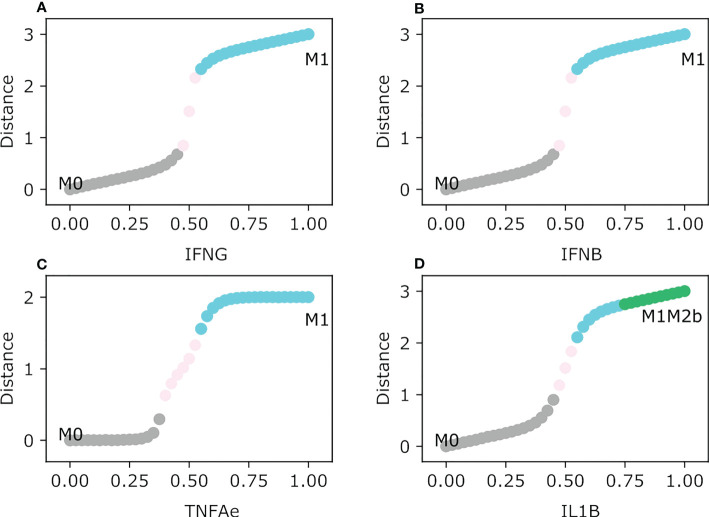
Macrophage Polarization in different Proinflammatory single exogenous cytokines. All panels have as an initial state the M0 (monocyte) phenotype. The plot shows in the y-axis the difference between the values of the initial state M0 and the final steady state, meanwhile the x-axis plots the gradual increase of the exogenous cytokines. **(A)** Gradual augmentation of the interferon-gamma (IFNG), **(B)** enhancement of the interferon-beta (IFNB), **(C)** gradual increase of the tumor necrosis factor-alpha (TNFAe), and **(D)** gradual augmentation of the interleukin 1-beta (IL1B).

To assess how concentrations of specific exogenous cytokines and metabolic byproducts shape macrophage polarization, we evaluated the feasible steady-state transition from the monocyte as a function of the concentration of the exogenous nodes (**Figure 2**). The first tested condition was the activation of cytokines involved in polarizing macrophages to a cytotoxic tumor-eliminating phenotype: IFNG, IFNB, TNFA, and IL1B. Interferon-γ and β polarized the monocyte to an M1 type macrophage where STAT1 is being specifically activated with a concentration of 0.55 for both types of interferons. From a theoretical point of view, our simulation allows us to note that there is a normalized range of concentration between 0.475 and 0.525, where the macrophage phenotype is not defined and we cannot describe its possible behavior (35) (**Figures 2A, B**). The M1 type STAT1 macrophage is important in a tumor microenvironment because of its connection with Th1 cells and the secretion of IL-12, a cytokine that maintains Th1 cells sustaining a tumor-eliminating microenvironment (36). A monocyte under TNFA follows the rise to an M1 type NFкB macrophage, but it will need a higher concentration of said factor roughly to a value of 0.6 to differentiate (**Figure 1C**). The M1-type NFкB macrophage is associated with secreting pro-inflammatory cytokines in tumor-eliminating factors (37). Nevertheless, an amount of pro-inflammatory cytokines may create a microenvironment inducing more damage than a solution, the M1 type NFкB macrophage has no regulation of the secretion of cytokines associated with the cytokine storm (38). The regulation may focus on the behavior of NFкB dynamics which is still an extensively studied transcription factor in macrophages (39).

IL1B induces the monocyte to differentiate into two types of macrophages based on its concentration. If the value of IL1B is relatively low, the monocyte will polarize to an M1 type NFкB/STAT1 type (**Figure 2D**). Moreover, it shifts to the hybrid macrophage phenotype M1M2b as IL1B is greater than 0.75. This hybrid can be an important component when the cytokine storm is triggered, it has the M2b phenotype as a counterpart involved in regulating inflammatory behavior produced by the M1 phenotype due to the activation of the ERK transcription factor (15). IL-6 has a dual role, it can activate an M1 AP-1 type macrophage and the activation of STAT3 which is conditioned by the presence of other cytokines. With a concentration of 0.45, it will abruptly jump from monocyte to an M1-type macrophage associated with the secretion of TNFA, which will be associated with a more inflammatory macrophage (**Figure 2C**). TNFA can become an inflection point in regulating the malignancy of a cytokine storm, by augmenting the secretion of pro-inflammatory cytokines and eliminating tumor cells by not allowing their division.

As **Figure 2** depicts some macrophage transitions, there is an interval in the graphs that displays NoLabel, we called this gap the range of uncertainty. It is a state where the macrophage phenotype is still not clearly defined by the model because the expression of a transcription factor is not unique to portray one of the considered phenotypes based on the cytokine in the microenvironment.

**Figure 3:**
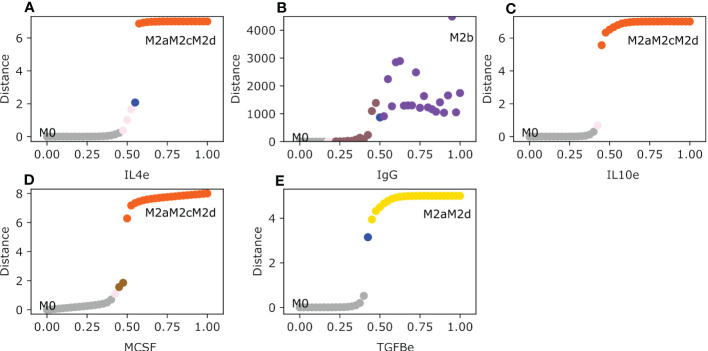
Macrophage Polarization in different Anti-Inflammatory single exogenous cytokines. All panels have as an initial state the M0 (monocyte) phenotype. The plot shows in the y-axis the difference between the values of the initial state M0 and the final steady state, meanwhile the x-axis plots the gradual increase of the exogenous cytokines. **(A)** Gradual augmentation of interleukin-4 (IL4e), **(B)** enhancement of immunoglobulin (IgG), **(C)** gradual increase of interleukin-10 (IL-10e), **(D)** gradual augmentation of Macrophage Colony Stimulating Factor (MCSF), and **(E)** gradual enhancement of tumor growth factor beta (TGFB).

The M2 macrophage subtypes (M2a, M2b, M2c, and M2d) are polarized by the following cytokines and metabolic byproducts: immune complexes (IgG), MCSF, TGFB, IL-10, IL-4, and adenosines. IL-4 activates the M2a macrophage phenotype, it is associated with inhibiting the Th1 response and enhancing the Th2 response (14). When the value of IL-4 is 0.55 the monocyte changes to an M2a phenotype for a short interval of time (**Figure 3A**). Because IL-4 is implicated in secreting IL-10 and TGFB in the microenvironment due to the activation of STAT6, the M2a phenotype jumps to the tumor-promoting macrophage phenotype M2aM2cM2d hybrid. The M2b macrophages need little value of the immune complexes (IgG) to induce the polarization from a monocyte to said phenotype. When IgG values are greater than 0.5 it favors the transition to the solo M2b phenotype after transiting in a brief time in the hybrid M2aM2bM2d macrophage (**Figure 3B**). IL-10 and TGFB induce tumor growth and alterations in the immune system, respectively. The range of uncertainty is low between 0.475 and 0.525. IL-10 can maintain the immune response at bay and prevents the elimination of tumor cells because it counterattacks the cytotoxic functions in macrophages (16). While IL10e≥0.45 , the monocyte will polarize to a hybrid phenotype M2aM2cM2d with a regulatory unfavorable behavior towards tumor promotion, and it will secrete growth factors and cytokines involved in regulating tumor elimination (**Figure 3C**). M2aM2cM2d develops a macrophage with only a regulatory capacity to inhibit T-cell expansion, reducing tumor cell clearance (40). So, IL-10 is a cytokine to be avoided in a tumor microenvironment because it is associated with a bad prognosis for any type of cancer, including breast cancer (41). MCSF is implicated in polarizing the monocyte to an M2-type phenotype. In a brief set of conditions when the value of MCSF is between 0.45 and 0.5, macrophages transit to an M2c phenotype. The phenotype stabilizes with a value greater than 0.5 to a pro-tumor hybrid M2aM2cM2d (**Figure 3D**). Meanwhile, adenosine expression in the microenvironment will make the monocyte transit between two phenotypes and stabilize to an M2aM2cM2d when the value is greater than 0.55. Between 0.5 and 0.52, it will first transit from M0 to an M2a phenotype, followed by the polarization to a hybrid phenotype M2aM2d with regulatory activity (**Figure S1**). Finally, TGFB will polarize monocytes to a regulatory hybrid M2aM2d, transiting first through a pure M2a macrophage when the values are between 0.425 and 0.525(**Figure 3E**). The M2d phenotype is associated with secreting vascular endothelial growth factor (it will help create new blood vessels) (37), mixed with the M2a compartment that will secrete TGFB that may enhance tumor growth, creating a perfect environment that will allow tumor cells to thrive.

Lastly, the continuous model of macrophage polarization in a tumor microenvironment sets the monocyte polarization shift given the tumor microenvironment when recruited. The transition between phenotypes is mostly gradual with a range of uncertainty but for some, the transition is abrupt. Hybrid phenotypes are stable steady states, meanwhile experimentally (M2a, M2b, M2c, and M2d) verified proven phenotypes are only phenotypes between the transition zones to a steady state.

### 3.2 Steady-state attractors evaluated in specific macrophage microenvironments

**Figure 4:**
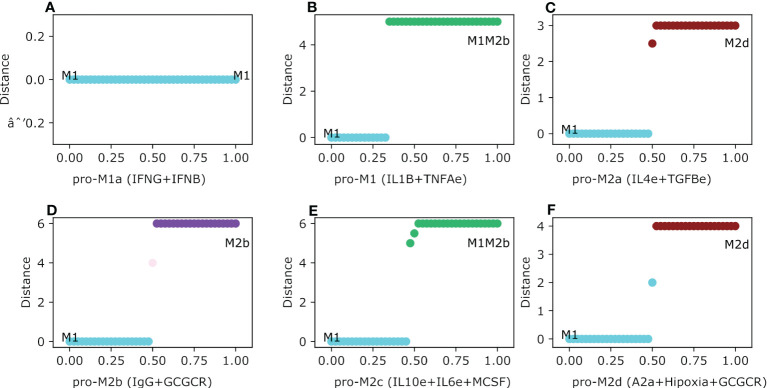
Macrophage Polarization in different phenotype-associated microenvironments. All panels have as an initial state the M0 (monocyte) phenotype. The plot shows in the y-axis the difference between the values of the initial state M0 and the final steady state, meanwhile the x-axis plots the gradual increase of the exogenous cytokines. **(A)** Gradual augmentation of the interferon-gamma (IFNG) and interferon-beta (IFNB) in the M1a environment, **(B)** enhancement of tumor necrosis factor-alpha (TNFAe) and interleukin 1-beta (IL1B) for the M1 microenvironment, **(C)** gradual increasing of interleukin-4 and tumor growth factor, a pro-M2a microenvironment, **(D)** gradual augmentation of immunoglobulin and glucocorticoids, the pro-M2b microenvironment, **(E)** gradual enhancement of interleukin-10, macrophage colony-stimulating factor and interleukin-6, the pro-M2c environment, and **(F)** gradual increase of adenosines, hypoxia and glucocorticoids, the pro-M2d environment.

In this section we will evaluate the stability of the thirteen intermediary steady-states of the macrophages theoretically obtained in our previous work (21) as a function of different microenvironments mimicking tumor space. Thus, having selected one steady-state (for example M1) we tested their robustness under different microenvironments present in a tumor microenvironment by gradually increasing the concentration of different cytokines. As a result, we arrived at the following conclusions. In agreement with previous reports, our model concludes that M1 macrophage is invariant under an interferon-present microenvironment, which means M1 maintains the same phenotype independent of interferon concentrations (**Figure 4A**). For the case of the cytotoxic macrophage M1, we evaluated the dynamics of the polarization based on adverse microenvironments conditions. First, when we gradually increased IL1B and TNFA simultaneously (a pro-cytokine storm microenvironment), M1 gets polarized and stabilized to a regulatory M1M2b hybrid phenotype. Interestingly, the macrophage changes its phenotype albeit the value for both factors was greater than 0.3 (**Figure 4B**). On the other hand, when we evaluated the M1-type macrophage in a regulatory microenvironment with IL4 and TGFB slightly expressed (**Figure 4C**), the phenotype changed to an M2d-type macrophage. Curiously, the M1 macrophage remained in the same phenotype until the external cytokine’s values were greater than 0.5. As the value of 0.5 is surpassed, the macrophage polarized to the M2 tumor-associated macrophage. IL-4 had no action on the activation of STAT6 because STAT1 was always present. Meanwhile, TGFB had the ability to activate HIF1α by inactivating STAT1. Therefore, this situation favors the development of a macrophage without cytotoxic capacities and with regulatory functionality. In the meantime, the M1 shifted to an M2b macrophage in the pro-M2b microenvironment. In this last case, an explanation can emerge by considering that the immune complexes mixed with glucocorticoids activate ERK and inactivate the cytotoxic function of STAT1 (**Figure 4D**). There is a slight moment of the interval range of uncertainty in this microenvironment when the value was between 0.5 and 0.525. By uncertainty, we mean a state where there is no way to label the phenotype of the macrophage in agreement with the classic biomarkers. The transition occurs when the value was greater than 0.525. On the other hand, the M1 macrophage coexisting in an immune regulatory microenvironment (pro-M2c) evolve to the M1M2b hybrid phenotype, which kept the cytotoxic counterpart of M1 mixed with M2b (**Figure 4E**). The polarization was set when the value of the external cytokines was higher than 0.5, and a range of uncertainty was observed in this simulation. Besides, if the M1 macrophage encounters a hypoxic microenvironment (which is very common in a tumor microenvironment), it will polarize into an M2-type macrophage (**Figure 4F**). As in previous cases, we noted a threshold in the concentration to proceed with the transition. The polarization takes place when values are bigger than 0.6 of hypoxia, glucocorticoid, and adenosine factors. Interestingly, when the values of these factors are scarce, the macrophage will remain in the cytotoxic behavior, allowing tumor elimination.

One of the hybrid phenotypes with promising therapeutic modification due to its cytotoxic/regulatory mechanisms is M1M2b. We assessed how this hybrid phenotype behaves in adverse microenvironments. Of the six environments evaluated (all experimentally proven in macrophages), only three were associated with a polarization scheme. In the other conditions, the macrophage maintains the same phenotype (**Figures S2A, B, E**). The pro-M2a condition was implicated in polarizing the M1M2b macrophage into an M2d macrophage, a regulator of the immune system (**Figure S2C**). This phenotype is implicated in maintaining a microenvironment that allows tumor cells to thrive. The M2d macrophage was also polarized in the pro-M2d microenvironment (**Figure S2F**). The M2b environment polarized the hybrid to solely M2b when the values were greater than 0.5 (**Figure S2D**).

We characterized previously, through a Boolean analysis of the transcriptional regulatory network of a macrophage, that the regulatory/pro-tumoral macrophage hybrid M2bM2d was the most stable attractor (it has a higher basin of attraction) (21). However, it behaved quite differently when introduced to specific environments, we found that only in two of six microenvironments the phenotype was maintained, the rest of the microenvironments encourage to polarize of the M2bM2d to another phenotype. For instance, the pro-M1a environment favors the polarization of the M2bM2d to M1M2b hybrid phenotype (**Figure S3A**). The interferon G and B had the ability to shift to a new phenotype when values were bigger than 0.1 and the phenotype stabilized as the interferon values were gradually increased. This hybrid state has the capacity to eliminate tumor cells due to the interferon secreted mixed with the regulatory component of the M2b macrophage. Overall, this condition simultaneously acts to eliminate and recover damaged tissue. Regarding the pro-M1 microenvironment (or the pro-cytokine storm environment), the M1M2b macrophage was not affected by the environment and stayed in the same initial phenotype (**Figure S3B**), whereas the M2bM2d is polarized to a different phenotype in the pro-M2a or wound-healing microenvironment. The hybrid macrophage polarizes the behavior to a completely regulatory/pro-tumor, named M2aM2d. An explanation of this fact is due to the action of external TGFB, which activates HIF1α and the STAT3 transcription factor (**Figure S3C**). This transition took place when the values of the external factors were bigger than 0.55. The M2b environment had interesting dynamic behavior when values of IgG and GCGCR were between 0.1 and 0.25 (**Figure S4D**). However, when we increased the values from 0.3 to 0.5, the macrophage went through a polarization toward the M2aM2bM2d phenotype, a regulatory hybrid state conformed by three phenotypes. In addition, the phenotype remains fixed to an M2bM2d macrophage with a regulatory/pro-tumoral environment. Meanwhile, the regulatory environment, pro-M2c, polarized the macrophage to a pro-tumoral phenotype and inactivated the immune system when external factors were greater than 0.2 (**Figure S3E**). This last type of microenvironment is common in tumors, and it is characterized by the presence of IL-10 and IL-6, which favors tumor proliferation and metastasis. On the other hand, the tumor core environment defined by the pro-M2d phenotype shifted the M2bM2d to the hybrid phenotype M2aM2cM2d, creating a scenario where tumor cells may thrive, and the macrophage can survive a complicated no-oxygen environment due to the M2a counterpart (**Figure S3F**).

Last, but not least important, we analyzed the pattern of behavior of the M2cM2d macrophage. This hybrid phenotype is implicated in tumor immune evasion because it has the ability to secrete IL-10. In addition, it promotes the secretion of factors that enhance tumor growth by creating new imperfect vessels with nutrients to reach the site where cancer cells are scarce. Accordingly, with our model, the interferon conditions had the ability to inactivate the M2c counterpart when values were bigger than 0.5, developing into an M2d macrophage. The pro-cytokine storm (**Figure S4B**) and the pro-M2a domain (**Figure S4C**) did not affect the behavior of the M2cM2d macrophage. Meanwhile, the M2b environment developed an interesting set of polarization schemes (**Figure S4D**). When the values of IgG and GCGCR were between 0.15 and 0.5 the M2cM2d polarized to an M2aM2cM2d. For a brief interval of time, the macrophage shifted to an M2aM2d, to finally converge on the M2aM2bM2d phenotype as the values were above 0.6. This phenotypic tri-hybrid macrophage can recuperate damaged tissue due to the activation of the inflammatory response, as the secretion of factors enhances tumor proliferation. Despite this, the microenvironment was able to inactivate the M2c phenotype, which may allow for the recruitment of macrophages with cytotoxic and tumor-eliminating functions. As expected, the pro-M2c environment did not affect the macrophage dynamics (**Figure S4E**). The hypoxic condition (pro-M2d) polarized the macrophage to an M2aM2cM2d hybrid. The M2a component was added to allow this macrophage to survive in an inhospitable microenvironment, enabling it to secrete factors to enhance tumor growth and create a hostile environment for tumor-eliminating cells (**Figure S4F**).

### 3.3 Theoretical genetically modified macrophages are resistant to specific macrophage phenotypes

**Figure 5:**
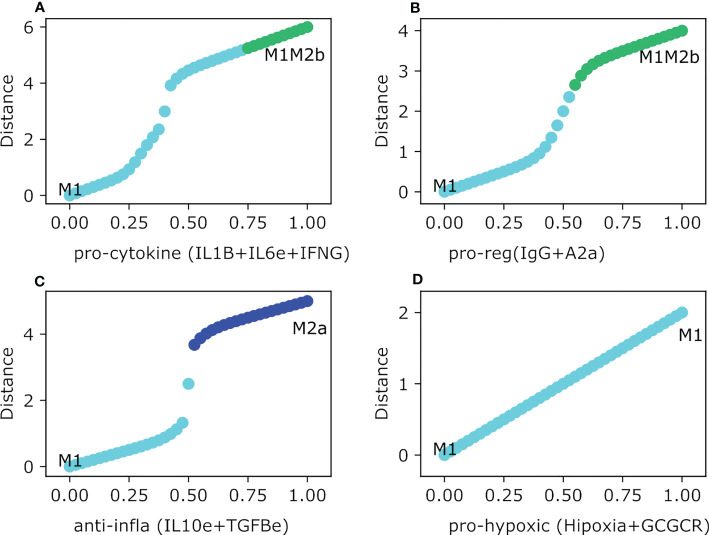
Theoretically Genetically Modified Macrophages (TGEM) in different Breast Cancer microenvironments. All panels have as an initial state the activation and inhibition as constant perturbations of NFкB and HIF1-α respectively. The plot shows in the y-axis the difference between the values of the initial state M0 and the final steady state, meanwhile the x-axis plots the gradual increase of the exogenous cytokines. **(A)** Gradual augmentation of interferon-gamma (IFNG), interleukin-beta (IL1B), and interleukin-6 in the pro-cytokine and tumor elimination environment, **(B)** enhancement of immunoglobulin-G **(IgG)** and adenosines (A2a), a pro-regulatory microenvironment, **(C)** gradual increasing of interleukin-10 and tumor growth factor, an anti-inflammatory microenvironment, **(D)** gradual augmentation of hypoxia and glucocorticoids, a pro-hypoxic microenvironment.

In our previous work, we developed a macrophage with the activation of NFкB and the inhibition of HIF1α to incline the balance from a protumoral over an anti-tumoral behavior. By targeting these transcription factors, the constructed regulatory network develops NFкB-activated M1 macrophages that secrete proinflammatory cytokines. Given previous results, the polarization can be shifted under specific conditions. Moreover, we wanted to evaluate the polarization dynamics in four different and simplified breast cancer microenvironments and evaluate if there is a theoretical pharmaceutical approach to deal with the tumor progression. We analyzed the effect on the following conditions: 1) IgG and Adenosines, 2) IL10 and TGFB, 3) IL1B, IL6, and IFNG, and 4) hypoxia with glucocorticoids. Of the four phenotypes obtained in the TGEM, only three developed a shift in the behavior of the phenotypes. The pro-cytokine storm and interferon microenvironment polarized from an M1 phenotype to a hybrid with regulatory components mixed with tumoricidal capacity. The shift was only achieved when the value was bigger than 0.75, creating a balance in the ability to eliminate tumor cells and recover the damage with the M2b counterpart (**Figure 5A**). The most complicated microenvironment was the one with the presence of IgG and A2a because it is implicated with metastasis. The M1 polarized to a hybrid phenotype M1M2b through a continuous transition. Because of the M1 compartment in the hybrid M1M2b, we will have the tumoricidal action of NFкB that can be associated with sufficient elimination of tumor cells. When values in the expression of IgG and adenosines are greater than 0.6, the macrophage polarizes to an M1M2b phenotype with tumor elimination (M1) mixed with the regulatory component (M2b) (**Figure 5B**). Despite this microenvironment associated with tumor metastasis (54), it generates a phenotype with a balance between anti-tumoral and pro-tumoral behaviors. IL10 and TGFB are the only microenvironments that shift the behavior to a pro-tumoral component. When levels of expression for the cytokines (IL10 and TGFB) are bigger than 0.6, the M1 phenotype polarizes to an M2a macrophage which will not create a suitable environment for tumor elimination (**Figure 5C**). Finally, the hypoxia and glucocorticoid environment does not affect all the transition of the macrophage. Thus, we concluded that despite the patterns of hypoxia is important in a tumor microenvironment (55) (**Figure 5D**).

### 3.4 The exogenous microenvironment affects the endogenous behavior of macrophage phenotypes plasticity

**Figure 6:**
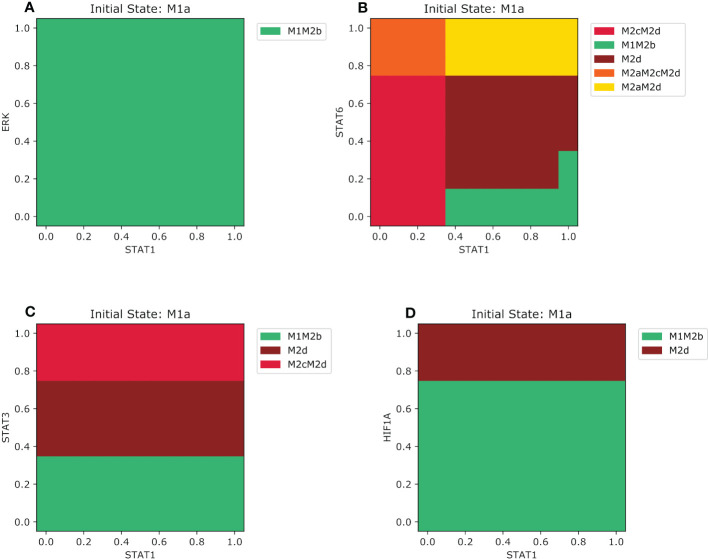
Phenotype space diagrams for the M1a macrophage phenotype in opposite microenvironments. All panels of the diagram have as an initial state the M1a macrophage (STAT1 activated). As well for all panels, STAT1 is gradually increased in the opposite macrophage microenvironments **(A)** Gradual augmentation of ERK transcription factor in a pro-M2b microenvironment, **(B)** enhancement of STAT6 transcription factor in a pro-M2a microenvironment **(C)** gradual increasing of the STAT3 transcription factor in a pro-M2c microenvironment, **(D)** gradual augmentation of HIF1-α in a pro-M2d microenvironment.

In the previous section, we have focused on how different environments influence the reversible transition between M1 and M2 types of macrophages. We concluded that only certain exogenous cytokines could change the behavior to polarize another phenotype based on the initial phenotypic states. This means that M1 and M2 phenotypes, and their hybrids stages, are not as plastic as we thought. In this section, we evaluated how the phenotypes behaved with initial lower concentrations of endogenous transcription factors of M1 and M2 subtype phenotypes. We evaluated these transcription factor concentrations in combination with their opposing cytokines (for example if we start with an M1 phenotype we will evaluate in IL-10 or TGFB environment) and studied the existence of partial polarization. As well, we assessed whether the qualitative concentration of a specific transcription factor is enough to stay in certain phenotypes or to shift to another. For instance, we selected the M1 phenotype when NFкB is activated and evaluated how certain level of expression of these transcription factors competes with the transcription factors activated when exposed to M2a, M2b, M2c, and M2d microenvironments. Our analysis allowed us to arrive at these conclusions. In an M2b microenvironment, the M1 will polarize to an M1M2b when values are greater than 0.35 of NFкB regardless of the value of ERK (**Figure S5A**). When values of NFкB are lower than 0.35 and ERK is lower than 0.2, the macrophages will shift back to the monocyte. When we increase the value of ERK between 0.2 and 0.75, there will be an interval where the macrophage cannot be labeled with a phenotype, in other words, the phenotype is still not determined. With an ERK greater than 0.75 and NFкB lower than 0.35, the M1 macrophage will polarize to a regulatory macrophage M2b. Overall, we observed that most of the time, the macrophage was in an anti-tumor/regulatory hybrid state. STAT6 (associated with an M2a phenotype) is much more dominant than ERK when we compare it with NFкB. When STAT6 relative concentrations are lower than 0.15, the M1 phenotype will maintain the same phenotype only if NFкB is lower than 0.35. for higher values, it will shift to an M1M2b regulatory/cytotoxic phenotype (**Figure S5B**). If STAT6 values are between 0.15 and 0.75 the M1 phenotype will transit to a pro-tumor M2d despite NFкB being present. However, if the STAT6 value is greater than 0.75 it will shift to a hybrid with wound-healing and regulatory behavior M2aM2d. The competition between the expression of STAT3 and NFкB is like STAT6. As STAT3 gets lower values than 0.35 the phenotype will transit to an M1M2b hybrid, and only if NFкB is greater than 0.35, lower values will maintain the same phenotype (**Figure S5C**). The complexity arises as STAT3 is greater than 0.35. For values between 0.45 and 0.75, the macrophage will shift to an M2aM2d hybrid allowing the activation of STAT6 by inhibiting the expression of NFкB. But for values greater than 0.75 and NFкB greater than 0.35 the macrophage will transit to a completely regulatory pro-tumoral macrophage M2aM2d creating a perfect environment for cancer to progress. HIF1α activation only inhibits a cytotoxic behavior if its value is greater than 0.75, polarizing the macrophage to an M2d phenotype. If HIF1α is lower than 0.75 the macrophage will behave as M1 or M1M2b phenotype based on the values of NFкB (**Figure S5D**). Instead, the M1 type activated by STAT1 behaves differently and maintains a certain dominance. For the M2b microenvironment regardless of the value of ERK, it will always be a shift to a hybrid M1M2b (**Figure 6A**). STAT6 and STAT1 have more competition when compared with STAT3 (**Figure 6B**). As STAT1 gets lower than 0.35 and STAT6 values are between 0 and 0.75 the macrophage will polarize to an M2cM2d regulatory of the immune system phenotype. But if the value of STAT1 is greater than 0.35 and STAT3 is lower than 0.15, the macrophage will polarize to a cytotoxic and regulatory hybrid M1M2d. When we combined higher values of STAT1 and a value of STAT6 between 0.15 and 0.75 the macrophage will transit to a pro-tumor macrophage M2d. Once STAT6 is greater than 0.75 we had two possible transitions based on the level of expression of STAT1. When values are lower than 0.35, it will shift to a three phenotypic hybrid with a completely pro-tumor behavior M2aM2cM2d, and when values are greater than 0.35 it will shift to an M2aM2d, inhibiting the action STAT3. STAT3 competitive interaction with STAT1 is based on the value of STAT3 (**Figure 6C**). For values lower than 0.35, the macrophage will polarize to an M1M2b, and for values between 0.35 and 0.75, it will polarize towards M2d. Values greater than 0.75 will shift the macrophage to a regulatory and pro-tumoral M2cM2d phenotype. STAT1 effect is more dominant versus HIF1α. Only ifHIF1α is greater than 0.75 the macrophage will polarize to an M2d phenotype, instead for lower values than 0.75 it will shift to an M1M2b macrophage (**Figure 6D**).

We compared the behavior of all the combinations of the M2 subtypes macrophages immerse into pro-M1 and all pro-M2 microenvironments. When the M2a macrophage is exposed to a pro-M1 microenvironment, the trajectory of the transition behaves slightly differently when we evaluate the M1 phenotype in the pro-M2a microenvironment. As normalized concentrations of STAT6 are lower than 0.35, regardless of the value of NFкB, the macrophage will shift to an anti-tumor/regulatory hybrid M1M2b (**Figure S6A**). Notably, once the concentration of STAT6 takes the values between 0.35 and 0.75, there is bistability based on the level of expression of NFкB. When NFкB is lower than 0.35 will shift the macrophage to an M2cM2d phenotype, meanwhile, greater values than 0.35 it will get polarized to M2d macrophage. Interestingly, our analysis concluded that the only way to maintain a hybrid phenotype with an M2a component is given values greater than 0.75. In this last situation, depending on the amount of NFкB, the M2 macrophage polarizes into M2aM2d and M2aM2cM2d. On the other hand, as the value of STAT6 is lower than 0.35, mixed with a variety of values of STAT1, we observe three types of behavior. First, at lower values of 0.25, the M2a will shift to a monocyte. Secondly, when STAT6 takes amounts between 0.25 and 0.75 there will be a range of uncertainty. Finally, when it takes values greater than 0.75 it will polarize to an M1 anti-tumor macrophage (**Figure S6B**). In addition, a STAT6 value between 0.35 and 0.75, regardless of the amount of STAT1, will polarize the M2a macrophage into M2d due to the secretion of TGFB. Higher values of STAT6 are associated with an M2aM2d hybrid phenotype. Moreover, the behavior changes if we introduced an M2a macrophage in an M2b microenvironment (**Figure S6C**). When STAT6 values are less than 0.35 and it is mixed with an ERK amount lower than 0.25, the macrophage will shift back to a monocyte, but for values of ERK between 0.25 and 0.75, the phenotype of the macrophage will not be defined. ERK values greater than 0.75 combined with low expression of STAT6, the macrophage will shift to an M2b phenotype (**Figure S6C**). As STAT6 is between 0.25 and 0.75, no matter the amount of NFкB, the macrophage will transit to M2d. STAT6 values greater than 0.75 are associated with a two-hybrid that includes the M2a phenotype. If values of ERK are lower than 0.75, then the M2a will shift to an M2aM2d phenotype, meanwhile values greater than 0.75 will include the M2b phenotype in a three phenotypic hybrid with regulatory behavior M2aM2bM2d macrophage.

M2b macrophage behaves differently when it is immersed in two types of M1 microenvironments. In the environment where NFкB has activated the M2b macrophage, it transits into two phenotypes based on the expression of ERK (**Figure S7A**). If ERK is greater than 0.45 the macrophage will polarize to an M2cM2d phenotype due to the secretion of regulatory cytokine IL-10, meanwhile, for lower values, it will shift to an M1M2b phenotype. In a STAT1-activated microenvironment, there is a gap of uncertainty as ERK and STAT1 are between 0.25 and 0.75 (**Figure S7B**). If STAT1 is higher than 0.75 the M2b macrophage will polarize to an M1 phenotype, in combination when ERK is lower than 0.75 but higher than 0.25. A combination of the higher value of STAT1 and ERK is associated with a hybrid M1M2b. With higher values of ERK mixed with lower values of STAT1, the macrophage will stay in the same phenotype. M2b in a STAT3 microenvironment has a complicated behavior (**Figure S7D**). When ERK values are between 0.25 and 0.75 mixed with a lower value of STAT3 there is a range of uncertainty. Regardless of the value of ERK, when the M2b is mixed with a range of STAT3 between 0.25 and 0.35, the macrophage shifts to an M2d phenotype. If the amount of STAT3 is between 0.45 and 0.75, combined with an ERK value lower than 0.75, its transits to an M2aM2d macrophage. Notably, higher values of STAT3 are associated with an M2aM2cM2d. When ERK is higher than 0.75 and STAT3 is higher than 0.35, M2b shifts to an M2aM2bM2d macrophage. Besides, lower values of STAT3 and a high amount of ERK are implicated that the macrophage remaining in an M2b phenotype (**Figure S7D**). In a hypoxic environment, the range of uncertainty is related to HIF1α and ERK lower than 0.45. In a hypoxic condition, M2a macrophage can appear for ERK values greater than 0.45, and HIF1α ranges between 0.45 and 0.75. If the HIF1α amount is greater than 0.75, there can be two possible phenotypes based on the expression of ERK (**Figure S7E**). For ERK lower than 0.75 the macrophage will polarize to an M2aM2cM2d macrophage, instead if ERK values are greater than 0.75 the macrophage shifts to an M2aM2bM2d phenotype, this last incorporating the M2b instead of the M2c counterpart (**Figure S7E**).

**Figure 7:**
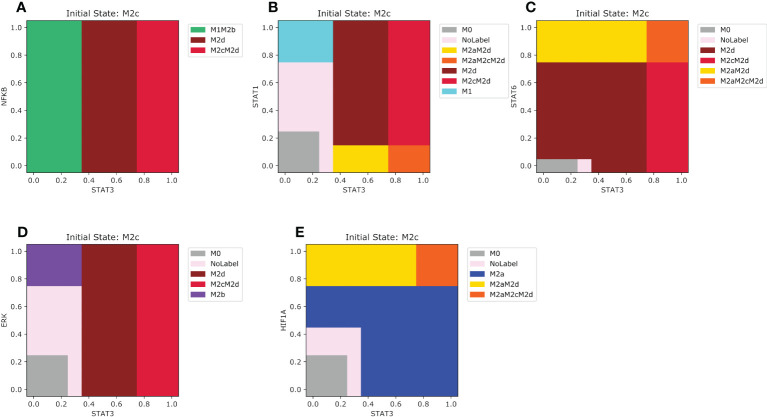
Phenotype space diagrams for the M2c macrophage phenotype in opposite microenvironments. All panels of the diagram have as an initial state the M2c macrophage (STAT3 activated). As well for all panels, STAT1 is gradually increased in the opposite macrophage microenvironments **(A)** Gradual increase of NFкB in a pro-M1 microenvironment, **(B)** Gradual enhancement of STAT1 in a pro-M1a microenvironment, **(C)** enhancement of STAT6 transcription factor in a pro-M2a microenvironment **(D)** Gradual augmentation of ERK transcription factor in a pro-M2b microenvironment, **(E)** gradual augmentation of HIF1-α in a pro-M2d microenvironment.

The most harmful effect of macrophage on health is in the M2c phenotype, which has prevailed over the M1 microenvironment. In the case of NFкB, if STAT3 is lower than 0.35 combined with an increasing value of NFкB (**Figure 7A**), the macrophage will shift to an M1M2b phenotype. Otherwise, if STAT3 is between 0.35 and 0.75, M2c will transit to an M2d macrophage. However, if STAT3 is greater than 0.75 regardless of the value of NFкB, M2c will polarize to an M2cM2d phenotype. For STAT1 the pattern of polarization for M2c is a little bit complex (**Figure 7B**). The macrophage may shift to an anti-tumoral phenotype if STAT1 is greater than 0.75 and STAT3 is lower than 0.35. In our analysis, we noted that STAT3 has a dominance against STAT1 if the value is greater than 0.35. In this last situation, the macrophage will transit to a pro-tumor and regulatory hybrid regardless of the expression of STAT3. When the M2c macrophage is in a microenvironment of tissue remodeling, when the values of STAT3 are between 0.35 and 0.65 and STAT6 are between 0.1 and 0.7 the macrophage will transit to a M2d phenotype with the capacity to regulate the growth of tumor cells (**Figure 7C**). For the M2b microenvironment STAT3 is only affected when the values of ERK are greater than 0.35 (**Figure 7D**). Greater values of ERK than 0.7 the macrophage will transit to a M2b phenotype (**Figure 7D**). Interestingly values of STAT3 lower than 0.35 and values of ERK between 0.25 and 0.7 the macrophage will have no label or a period uncertainty (**Figure 7D**). In a hypoxic microenvironment, the M2a phenotype dominates in the polarization dynamics (**Figure 7E**). Only when STAT3 is higher than 0.75 the macrophage will shift to a hybrid M2aM2cM2d, a hybrid phenotype that simultaneously includes the M2c and M2a phenotypes. The M2d macrophage converges to different phenotypes when exposed to M1 types of microenvironments.

In brief, to understand the overall dynamics of the possible phenotypes of macrophages in a tumor microenvironment, we must focus not only on the cytokines present but also on the concentration of the transcription factors. Nevertheless, it is not only enough to know the concentration, but also the mechanisms of direct interaction with other transcriptional factors. Combining all layers of information, we can unravel the complexity of macrophage adaptation in a continuously changing environment and try to predict possible behavioral changes. Altogether, we highlight that this analysis can serve as a framework for designing testable experimental strategies that prevent detrimental phenotypes to the patient.

## 4 Discussion

The interaction between the immune system, the microenvironment, and cancer is one appealing topic to design effective treatment strategies. To do so, we need to understand how cancer and immune signals affect the behavior of endogenous factors in macrophage polarization in a continuum approach. The analysis of our mathematical model demonstrates contradictory patterns of differentiation of macrophages under different microenvironments. Even more challenging and counter-intuitive, the complexity increase when we take into account the combination of cytokines present in a tumor microenvironment. As expected, the pattern of macrophage differentiation is shaped by the regulatory and signaling network whose components and circuits eventually support the pro- and anti-inflammatory responses against cancer cells. In our model, we demonstrated that exogenous cytokines and endogenous components are crucial for macrophage functional behavior and their emerging phenotypes (36).Thus, our mathematical approach can study the transition between phenotypes based on an initial state and a combination of exogenous cytokines. In a glimpse, we observed two types of transitions between macrophage phenotypes: continuous and discontinuous. Discontinuous transitions occur when the change between the initial state and steady states has a sudden shift, once we overcome a threshold in the intensity of an external signal, see for instance **Figure 4B**. Meanwhile, continuous transitions comprise those trajectories that occur in a gradual fashion, for instance, see **Figure 5E**. Notably, continuous transition was only present in the M1 subtypes with an uncertainty region where both M1 transcription factors, NFкB and STAT1, were activated but not enough to develop in a differentiated macrophage with functional interferon or pro-inflammatory response. The uncertainty came from the poor knowledge about macrophage polarization, despite having explored and described intermediate states, results pointed out the existence of other macrophage responses. Instead, we may have a range of functional intermediate behavior of a specific molecule that can coexist or change to one another under certain microenvironments. This regulation of cytokine behavior is observed in response to interferons, TNF-α, and IL-1β. The regulatory effects of said exogenous cytokines will dictate the type of cytotoxic behavior based on the microenvironment. It is not the same response based on interferon or interleukins (56, 57). Exogenous cytokines may be used as potential immunotherapy due to their immunoregulatory behavior, but they are pleiotropy and have poor-like drug properties. So, we can obtain a macrophage with anti-tumor properties mixed with pro-inflammatory cytokines like the range of expression in M1 macrophages (58).

Our model recognize the importance of cytokines when they act as single or in cooperation with other signals. For the M1 type macrophage, a single cytokine had the sufficient capacity to polarize the monocyte to the M1 state. Nevertheless, when the synergy occurs in specific environments, it produced an acceleration of activation compared with the activation with a single cytokine. Based on our model, this synergy of signals has the capacity to activate macrophages with hybrid phenotypes (with at least two types of macrophage behavior). For example, M1M2b and M1M2d hybrid states emerge mainly through their interaction with the microenvironment (For example IgG, IL1β and TNF-α). Notably, these M1-like tumor-associated macrophages (TAM) have dual or bi-directional behavior, these have the capacity to eliminate and promote the motility of tumor cells simultaneously (59, 60). A similar antagonist effect may happen with M1M2d state, which can eliminate tumor cells, promote angiogenesis or metastasis depending of the signal profiles (60).The same phenotypic responses of having a dual response were observed for the hybrids that only include M2 subtypes. This being said, the hybrids will have a plethora of functions that go from regulating the immune response, promoting angiogenesis to tissue remodeling. The hybrid states falling into this category are: M2aM2d, M2bM2d, and M2cM2d (61–63).

In addition, we evaluated the plasticity of the response of the M1 and the M2 subtypes in opposing cytokine microenvironments. We conclude that once M1 or M2 reaches the steady state, it will be difficult to transit toward other phenotypes, even exposing them to opposing microenvironments. On the other hand, the plasticity of partially polarized macrophages (hybrid states) is quite different. Based on the expression of certain transcription factors, it seems that macrophages in hybrid phenotypes can transit more easily between phenotypes, and the transition is mainly affected by the microenvironment of the opposing cytokine.

Finally, our mathematical approach has some limitations and challenges that should be addressed in future works. Even though we have included the time as a continuous variable, the model is circumscribed to build qualitative hypotheses of the transition of macrophage phenotypes. As a consequence, we cannot obtain the precise dynamics of the transition but only the qualitative behavior that it portrays. Besides, in our study, some biological and physical factors have been neglected for the sake of simplicity. For instance, it neglected the influence of the extracellular matrix in shaping the macrophage polarization by inhibiting the integrins in the surface of the membrane of macrophages. In addition, the diffusion of signals is absent, which in real cases is fundamental to analyzing the heterogeneous macrophage phenotype in space and time. A careful analysis of these factors on macrophages phenotypes is an interesting question that can be addressed in future studies. On the other hand, one of the outstanding challenges to overcome is the experimental validation of in-silico observations. We expect that this theoretical model mixed with recent experimental evidence may be used as a tool to understand, design, and explain the diversity of macrophages in a tumor microenvironment. Undoubtedly, the positive crosstalk between experimental results and theoretical models is a valuable enterprise to unravel the complexity of the function of macrophages in a tumor microenvironment and develop experimental strategies for maintaining the functional homeostasis between pro- and anti-inflammatory disrupted by cancer disease (64). This being said, we can integrate the description of tumor associated macrophages (TAMs) using single-cell technologies with the theoretical outcomes obtained in this in-silico work or others to solve the unification of the TAMs subsets in tumor microenvironments. Undoubtedly, this aim will be a new avenue to transit in future studies.

Finally, an important aim to look at in the future is to evaluate the possible clinical relevance of the obtained phenotypes. In our previous work (21), we theoretically postulated some hybrid phenotypes with a tumoricidal capacity or promoting regulatory mechanisms against cancer. These preditions claims for experimental assessment. For instance, we concluded that our tumor-eliminating hybrids (M1M2d and M1M2b) behave similarly to the interferon-primed tumor-associated macrophages (TAMs) and the inflammatory cytokines enrich TAMs (65). Interestingly, both types of TAMs have been characterized through single-cell technologies. So, in order to understand and solve the unification of the TAM subset of all possible tumor microenvironments, it is desirable to integrate theoretical solutions with single-cell data. Undoubtedly, this aim will be a new avenue to transit in future studies.

## Data availability statement

The original contributions presented in the study are included in the article/**Supplementary Materials**, further inquiries can be directed to the corresponding author/s.

## Author contributions

UA-P and OR-A conceived and designed the mathematical model. UA-P and AV-J performed all computational analyses and analyzed the data. AV-J helped with the analyses of the data. PP-L and OR-A supervised the mathematical model and the in-silico analysis. All authors contributed to the article and approved the submitted version.

## References

[1] Palma A Jarrah AS Tieri P Cesareni G Castiglione F. Gene regulatory network modeling of macrophage differentiation corroborates the continuum hypothesis of polarization states. Front Physiol (2018) 9:1659. doi: 10.3389/fphys.2018.01659 30546316 PMC6278720

[2] Ramirez R Herrera AM Ramirez J Qian C Melton DW Shireman PK. Deriving a boolean dynamics to reveal macrophage activation with *in vitro* temporal cytokine expression profiles. BMC Bioinf (2019) 20:725. doi: 10.1186/s12859-019-3304-5 PMC6921543 31852428

[3] Ordaz-Arias MA Díaz-Alvarez L Zúñiga J Martinez-Sánchez ME Balderas-Martínez YI. Cyclic attractors are critical for macrophage differentiation, heterogeneity, and plasticity. Front Mol Biosci (2022) 9:807228. doi: 10.3389/fmolb.2022.807228 35480895 PMC9035596

[4] Marku M Verstraete N Raynal F Madrid-Mencía M Domagala M Fournié J-J. Insights on TAM formation from a boolean model of macrophage polarization based on *In vitro* studies. Cancers (2020) 12:1–23. doi: 10.3390/cancers12123664 PMC7762229 33297362

[5] Vázquez-Jiménez A Avila-Ponce De León UE Matadamas-Guzman M Muciño-Olmos EA Martínez-López YE Escobedo-Tapia T. On deep landscape exploration of COVID-19 patients cells and severity markers. Front Immunol (2021) 12:705646. doi: 10.3389/fimmu.2021.705646 34603282 PMC8481922

[6] Deligne C Midwood KS. Macrophages and extracellular matrix in breast cancer: partners in crime or protective allies? Front Oncol (2021) 11:620773. doi: 10.3389/fonc.2021.620773 33718177 PMC7943718

[7] Kumar V. Macrophages: the potent immunoregulatory innate immune cells. Macrophage Act -Biol Dis (2019). doi: 10.5772/intechopen.88013

[8] Italiani P Boraschi D. From monocytes to M1/M2 macrophages: phenotypical vs. functional differentiation. Front Immunol (2014) 5:514. doi: 10.3389/fimmu.2014.00514 25368618 PMC4201108

[9] Arango Duque G Descoteaux A. Macrophage cytokines: involvement in immunity and infectious diseases. Front Immunol (2014) 5:491. doi: 10.3389/fimmu.2014.00491 25339958 PMC4188125

[10] Piccolo V Curina A Genua M Ghisletti S Simonatto M Sabò A. Opposing macrophage polarization programs show extensive epigenomic and transcriptional cross-talk. Nat Immunol (2017) 18:530–40. doi: 10.1038/ni.3710 PMC5524187 28288101

[11] Wang T Liu G Wang R. The intercellular metabolic interplay between tumor and immune cells. Front Immunol (2014) 5:358. doi: 10.3389/fimmu.2014.00358 25120544 PMC4112791

[12] Nagarsheth N Wicha MS Zou W. Chemokines in the cancer microenvironment and their relevance in cancer immunotherapy. Nat Rev Immunol (2017) 17:559–72. doi: 10.1038/nri.2017.49 PMC5731833 28555670

[13] Biswas SK Sica A Lewis CE. Plasticity of macrophage function during tumor progression: regulation by distinct molecular mechanisms. J Immunol (2008) 180:2011–7. doi: 10.4049/jimmunol.180.4.2011 18250403

[14] Van Dyken SJ Locksley RM. Interleukin-4- and interleukin-13-mediated alternatively activated macrophages: roles in homeostasis and disease. Annu Rev Immunol (2013) 31:317–43. doi: 10.1146/annurev-immunol-032712-095906 PMC3606684 23298208

[15] Wang L-X Zhang S-X Wu H-J Rong X-L Guo J. M2b macrophage polarization and its roles in diseases. J Leukoc Biol (2019) 106:345–58. doi: 10.1002/JLB.3RU1018-378RR PMC7379745 30576000

[16] Martinez FO Gordon S. The M1 and M2 paradigm of macrophage activation: time for reassessment. F1000Prime Rep (2014) 6:13. doi: 10.12703/P6-13 24669294 PMC3944738

[17] Biswas SK Gangi L Paul S Schioppa T Saccani A Sironi M. A distinct and unique transcriptional program expressed by tumor-associated macrophages (defective NF-kappaB and enhanced IRF-3/STAT1 activation). Blood (2006) 107:2112–22. doi: 10.1182/blood-2005-01-0428 16269622

[18] Wang Q Ni H Lan L Wei X Xiang R Wang Y. Fra-1 protooncogene regulates IL-6 expression in macrophages and promotes the generation of M2d macrophages. Cell Res (2010) 20:701–12. doi: 10.1038/cr.2010.52 20386569

[19] Duluc D Delneste Y Tan F Moles M-P Grimaud L Lenoir J. Tumor-associated leukemia inhibitory factor and IL-6 skew monocyte differentiation into tumor-associated macrophage-like cells. Blood (2007) 110:4319–30. doi: 10.1182/blood-2007-02-072587 17848619

[20] Liu SX Gustafson HH Jackson DL Pun SH Trapnell C. Trajectory analysis quantifies transcriptional plasticity during macrophage polarization. Sci Rep (2020) 10:12273. doi: 10.1038/s41598-020-68766-w 32703960 PMC7378057

[21] Avila-Ponce de León U Vázquez-Jiménez A Matadamas-Guzman M Pelayo R Resendis-Antonio O. Transcriptional and microenvironmental landscape of macrophage transition in cancer: a boolean analysis. Front Immunol (2021) 12:642842. doi: 10.3389/fimmu.2021.642842 34177892 PMC8222808

[22] Das A Yang C-S Arifuzzaman S Kim S Kim SY Jung KH. High-resolution mapping and dynamics of the transcriptome, transcription factors, and transcription Co-factor networks in classically and alternatively activated macrophages. Front Immunol (2018) 9:22. doi: 10.3389/fimmu.2018.00022 29403501 PMC5778122

[23] Xue J Schmidt SV Sander J Draffehn A Krebs W Quester I. Transcriptome-based network analysis reveals a spectrum model of human macrophage activation. Immunity (2014) 40:274–88. doi: 10.1016/j.immuni.2014.01.006 PMC3991396 24530056

[24] Hickman E Smyth T Cobos-Uribe C Immormino R Rebuli ME Moran T. Expanded characterization of *in vitro* polarized M0, M1, and M2 human monocyte-derived macrophages: bioenergetic and secreted mediator profiles. PloS One (2023) 18:e0279037. doi: 10.1371/journal.pone.0279037 36862675 PMC9980743

[25] El Kasmi KC Stenmark KR. Contribution of metabolic reprogramming to macrophage plasticity and function. Semin Immunol (2015) 27:267–75. doi: 10.1016/j.smim.2015.09.001 PMC4677817 26454572

[26] Sheu KM Guru AA Hoffmann A. Quantifying stimulus-response specificity to probe the functional state of macrophages. Cell Syst (2023) 14:180–195.e5. doi: 10.1016/j.cels.2022.12.012 36657439 PMC10023480

[27] Yan C Li K Meng F Chen L Zhao J Zhang Z. Integrated immunogenomic analysis of single-cell and bulk tissue transcriptome profiling unravels a macrophage activation paradigm associated with immunologically and clinically distinct behaviors in ovarian cancer. J Advert Res (2023) 44:149–60. doi: 10.1016/j.jare.2022.04.006 PMC9936412 36725186

[28] Tiemeijer BM Heester S Sturtewagen AYW Smits AIPM Tel J. Single-cell analysis reveals TLR-induced macrophage heterogeneity and quorum sensing dictate population wide anti-inflammatory feedback in response to LPS. Front Immunol (2023) 14:1135223. doi: 10.3389/fimmu.2023.1135223 36911668 PMC9998924

[29] Yang K Yang T Yu J Li F Zhao X. Integrated transcriptional analysis reveals macrophage heterogeneity and macrophage-tumor cell interactions in the progression of pancreatic ductal adenocarcinoma. BMC Cancer (2023) 23:199. doi: 10.1186/s12885-023-10675-y 36864399 PMC9983236

[30] O’Carroll C Fagan A Shanahan F Carmody RJ. Identification of a unique hybrid macrophage-polarization state following recovery from lipopolysaccharide tolerance. J Immunol (2014) 192:427–36. doi: 10.4049/jimmunol.1301722 24337373

[31] Hagemann T Wilson J Burke F Kulbe H Li NF Plüddemann A. Ovarian cancer cells polarize macrophages toward a tumor-associated phenotype. J Immunol (2006) 176:5023–32. doi: 10.4049/jimmunol.176.8.5023 16585599

[32] Ramírez C Mendoza L. Phenotypic stability and plasticity in GMP-derived cells as determined by their underlying regulatory network. Bioinformatics (2018) 34:1174–82. doi: 10.1093/bioinformatics/btx736 29186334

[33] Smith TD Tse MJ Read EL Liu WF. Regulation of macrophage polarization and plasticity by complex activation signals. Integr Biol (2016) 8:946–55. doi: 10.1039/c6ib00105j PMC5148158 27492191

[34] Nickaeen N Ghaisari J Heiner M Moein S Gheisari Y. Agent-based modeling and bifurcation analysis reveal mechanisms of macrophage polarization and phenotype pattern distribution. Sci Rep (2019) 9:12764. doi: 10.1038/s41598-019-48865-z 31484958 PMC6726649

[35] Zhao C Mirando AC Sové RJ Medeiros TX Annex BH Popel AS. A mechanistic integrative computational model of macrophage polarization: implications in human pathophysiology. PloS Comput Biol (2019) 15:e1007468. doi: 10.1371/journal.pcbi.1007468 31738746 PMC6860420

[36] Frank AS Larripa K Ryu H Snodgrass RG Röblitz S. Bifurcation and sensitivity analysis reveal key drivers of multistability in a model of macrophage polarization. J Theor Biol (2021) 509:110511. doi: 10.1016/j.jtbi.2020.110511 33045246

[37] Villarreal C Padilla-Longoria P Alvarez-Buylla ER. General theory of genotype to phenotype mapping: derivation of epigenetic landscapes from n-node complex gene regulatory networks. Phys Rev Lett (2012) 109:118102. doi: 10.1103/PhysRevLett.109.118102 23005679

[38] Davila-Velderrain J Villarreal C Alvarez-Buylla ER. Reshaping the epigenetic landscape during early flower development: induction of attractor transitions by relative differences in gene decay rates. BMC Syst Biol (2015) 9:20. doi: 10.1186/s12918-015-0166-y 25967891 PMC4438470

[39] Kawasaki T Kawai T. Toll-like receptor signaling pathways. Front Immunol (2014) 5:461. doi: 10.3389/fimmu.2014.00461 25309543 PMC4174766

[40] Malyshev I Malyshev Y. Current concept and update of the macrophage plasticity concept: intracellular mechanisms of reprogramming and M3 macrophage “Switch” phenotype. BioMed Res Int (2015) 2015:1–23. doi: 10.1155/2015/341308 PMC4561113 26366410

[41] Orecchioni M Ghosheh Y Pramod AB Ley K. Macrophage polarization: different gene signatures in M1(LPS+) vs. classically and M2(LPS-) vs. alternatively activated macrophages. Front Immunol (2019) 10:1084. doi: 10.3389/fimmu.2019.01084 31178859 PMC6543837

[42] Ruffell B Affara NI Coussens LM. Differential macrophage programming in the tumor microenvironment. Trends Immunol (2012) 33:119–26. doi: 10.1016/j.it.2011.12.001 PMC3294003 22277903

[43] Piersma B Hayward M-K Weaver VM. Fibrosis and cancer: a strained relationship. Biochim Biophys Acta Rev Cancer (2020) 1873:188356. doi: 10.1016/j.bbcan.2020.188356 32147542 PMC7733542

[44] Kratochvill F Neale G Haverkamp JM Van de Velde L-A Smith AM Kawauchi D. TNF counterbalances the emergence of M2 tumor macrophages. Cell Rep (2015) 12:1902–14. doi: 10.1016/j.celrep.2015.08.033 PMC4581986 26365184

[45] Arora S Dev K Agarwal B Das P Syed MA. Macrophages: their role, activation and polarization in pulmonary diseases. Immunobiology (2018) 223:383–96. doi: 10.1016/j.imbio.2017.11.001 PMC7114886 29146235

[46] Bally APR Lu P Tang Y Austin JW Scharer CD Ahmed R. NF-κB regulates PD-1 expression in macrophages. J Immunol (2015) 194:4545–54. doi: 10.4049/jimmunol.1402550 PMC4402259 25810391

[47] Rhee I. Diverse macrophages polarization in tumor microenvironment. Arch Pharm Res (2016) 39:1588–96. doi: 10.1007/s12272-016-0820-y 27562774

[48] Ohama H Asai A Ito I Suzuki S Kobayashi M Higuchi K. M2b macrophage elimination and improved resistance of mice with chronic alcohol consumption to opportunistic infections. Am J Pathol (2015) 185:420–31. doi: 10.1016/j.ajpath.2014.09.022 25485859

[49] Wang H Wang L Chi P-D Wang W Chen X-Q Geng Q-R. High level of interleukin-10 in serum predicts poor prognosis in multiple myeloma. Br J Cancer (2016) 114:463–8. doi: 10.1038/bjc.2016.11 PMC4815778 26882069

[50] Zhao S Wu D Wu P Wang Z Huang J. Serum IL-10 predicts worse outcome in cancer patients: a meta-analysis. PloS One (2015) 10:e0139598. doi: 10.1371/journal.pone.0139598 26440936 PMC4595202

[51] Hutchins AP Diez D Miranda-Saavedra D. The IL-10/STAT3-mediated anti-inflammatory response: recent developments and future challenges. Brief Funct Genomics (2013) 12:489–98. doi: 10.1093/bfgp/elt028 PMC3838198 23943603

[52] Duluc D Corvaisier M Blanchard S Catala L Descamps P Gamelin E. Interferon-gamma reverses the immunosuppressive and protumoral properties and prevents the generation of human tumor-associated macrophages. Int J Cancer (2009) 125:367–73. doi: 10.1002/ijc.24401 19378341

[53] Anders CB Lawton TMW Smith HL Garret J Doucette MM Ammons MCB. Use of integrated metabolomics, transcriptomics, and signal protein profile to characterize the effector function and associated metabotype of polarized macrophage phenotypes. J Leukoc Biol (2022) 111:667–93. doi: 10.1002/JLB.6A1120-744R PMC8825884 34374126

[54] Cui M Huang J Zhang S Liu Q Liao Q Qiu X. Immunoglobulin expression in cancer cells and its critical roles in tumorigenesis. Front Immunol (2021) 12:613530. doi: 10.3389/fimmu.2021.613530 33841396 PMC8024581

[55] Michiels C Tellier C Feron O. Cycling hypoxia: a key feature of the tumor microenvironment. Biochim Biophys Acta (2016) 1866:76–86. doi: 10.1016/j.bbcan.2016.06.004 27343712

[56] Lee S Margolin K. Cytokines in cancer immunotherapy. Cancers (2011) 3:3856–93. doi: 10.3390/cancers3043856 PMC3763400 24213115

[57] Holder PG Lim SA Huang CS Sharma P Dagdas YS Bulutoglu B. Engineering interferons and interleukins for cancer immunotherapy. Adv Drug Delivery Rev (2022) 182:114112. doi: 10.1016/j.addr.2022.114112 35085624

[58] Briukhovetska D Dörr J Endres S Libby P Dinarello CA Kobold S. Interleukins in cancer: from biology to therapy. Nat Rev Cancer (2021) 21:481–99. doi: 10.1038/s41568-021-00363-z PMC8173513 34083781

[59] Wang H Wang X Li X Fan Y Li G Guo C. CD68(+)HLA-DR(+) M1-like macrophages promote motility of HCC cells *via* NF-κB/FAK pathway. Cancer Lett (2014) 345:91–9. doi: 10.1016/j.canlet.2013.11.013 24333724

[60] Xiao M Zhang J Chen W Chen W. M1-like tumor-associated macrophages activated by exosome-transferred THBS1 promote malignant migration in oral squamous cell carcinoma. J Exp Clin Cancer Res (2018) 37:143. doi: 10.1186/s13046-018-0815-2 29986759 PMC6038304

[61] Liu KX Joshi S. “Re-educating” tumor associated macrophages as a novel immunotherapy strategy for neuroblastoma. Front Immunol (2020) 11:1947. doi: 10.3389/fimmu.2020.01947 32983125 PMC7493646

[62] Fu L-Q Du W-L Cai M-H Yao J-Y Zhao Y-Y Mou X-Z. The roles of tumor-associated macrophages in tumor angiogenesis and metastasis. Cell Immunol (2020) 353:104119. doi: 10.1016/j.cellimm.2020.104119 32446032

[63] Hwang I Kim JW Ylaya K Chung EJ Kitano H Perry C. Tumor-associated macrophage, angiogenesis and lymphangiogenesis markers predict prognosis of non-small cell lung cancer patients. J Transl Med (2020) 18:443. doi: 10.1186/s12967-020-02618-z 33228719 PMC7686699

[64] Pittet MJ Michielin O Migliorini D. Clinical relevance of tumour-associated macrophages. Nat Rev Clin Oncol (2022) 19:402–21. doi: 10.1038/s41571-022-00620-6 35354979

[65] Ma R-Y Black A Qian B-Z. Macrophage diversity in cancer revisited in the era of single-cell omics. Trends Immunol (2022) 43:546–63. doi: 10.1016/j.it.2022.04.008 35690521

