# Supplementary material for: Uncoding the interdependency of tumor microenvironment and macrophage polarization: insights from a continuous network approach
Source: Front Immunol. 2023 May 22;14:1150890. doi: 10.3389/fimmu.2023.1150890 (PMC10240616; doi:10.3389/fimmu.2023.1150890)
Supplement: Supplementary file 1 [file DataSheet_1.docx]

Supplementary Material

Uncoding the Interdependency of Tumor Microenvironment and Macrophage Polarization: Insights from a Continuous Network Approach

**Ugo Avila-Ponce de León^1,2^, Aarón Vázquez-Jiménez^2^, Pablo Padilla-Longoria^3^ and Osbaldo Resendis-Antonio^2,4,5*^**

^1^Programa de Doctorado en Ciencias Biológicas, Universidad Nacional Autónoma de México, Ciudad de México, México.

^2^Human Systems Biology Laboratory, Instituto Nacional de Medicina Genómica (INMEGEN), Ciudad de México, México.

^3^Institute for Applied Mathematics (IIMAS), Universidad Nacional Autónoma de México, Ciudad de México, México.

^4^Coordinación de la Investigación Científica - Red de Apoyo a la Investigación, UNAM, Ciudad de México, México.

^5^Centro de Ciencias de la Complejidad (C3), Universidad Nacional Autónoma de México, Ciudad de México, México.

*** Correspondence:**

**Ugo Avila-Ponce de Leon**

[ugo.avila.ponce@gmail.com](mailto:ugo.avila.ponce@gmail.com)

**Dr. Osbaldo Resendis-Antonio**
oresendis@inmegen.gob.mx

# Supplementary Figures


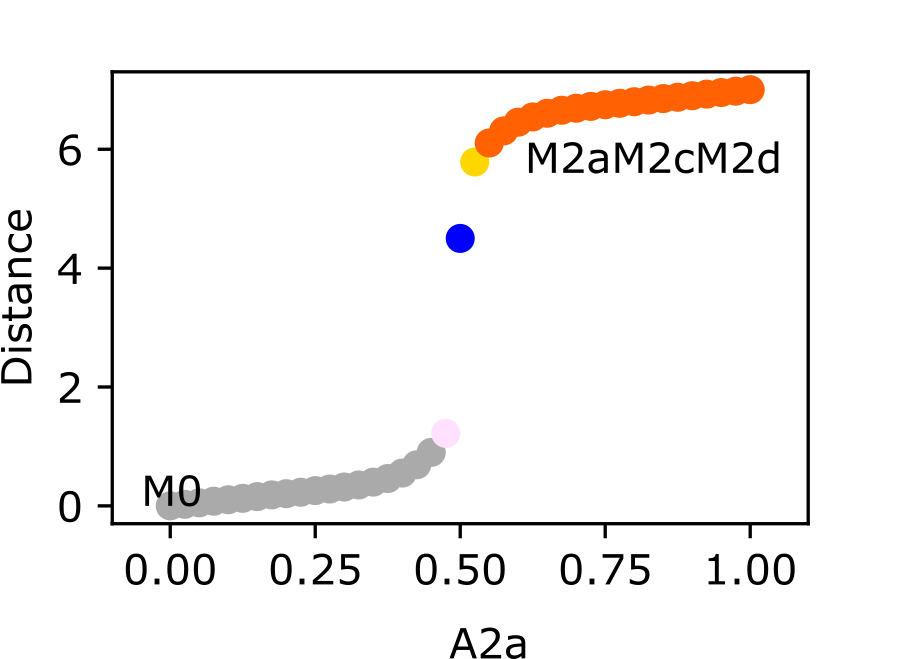


### Figure S1. Macrophage Polarization in different single exogenous cytokines. All panels have as an initial state the M0 (monocyte) phenotype. The plot shows in the y-axis the difference between the values of the initial state M0 and the final steady-state, meanwhile the x-axis plots the gradual increase of the exogenous cytokines. Gradual augmentation of Adenosines.


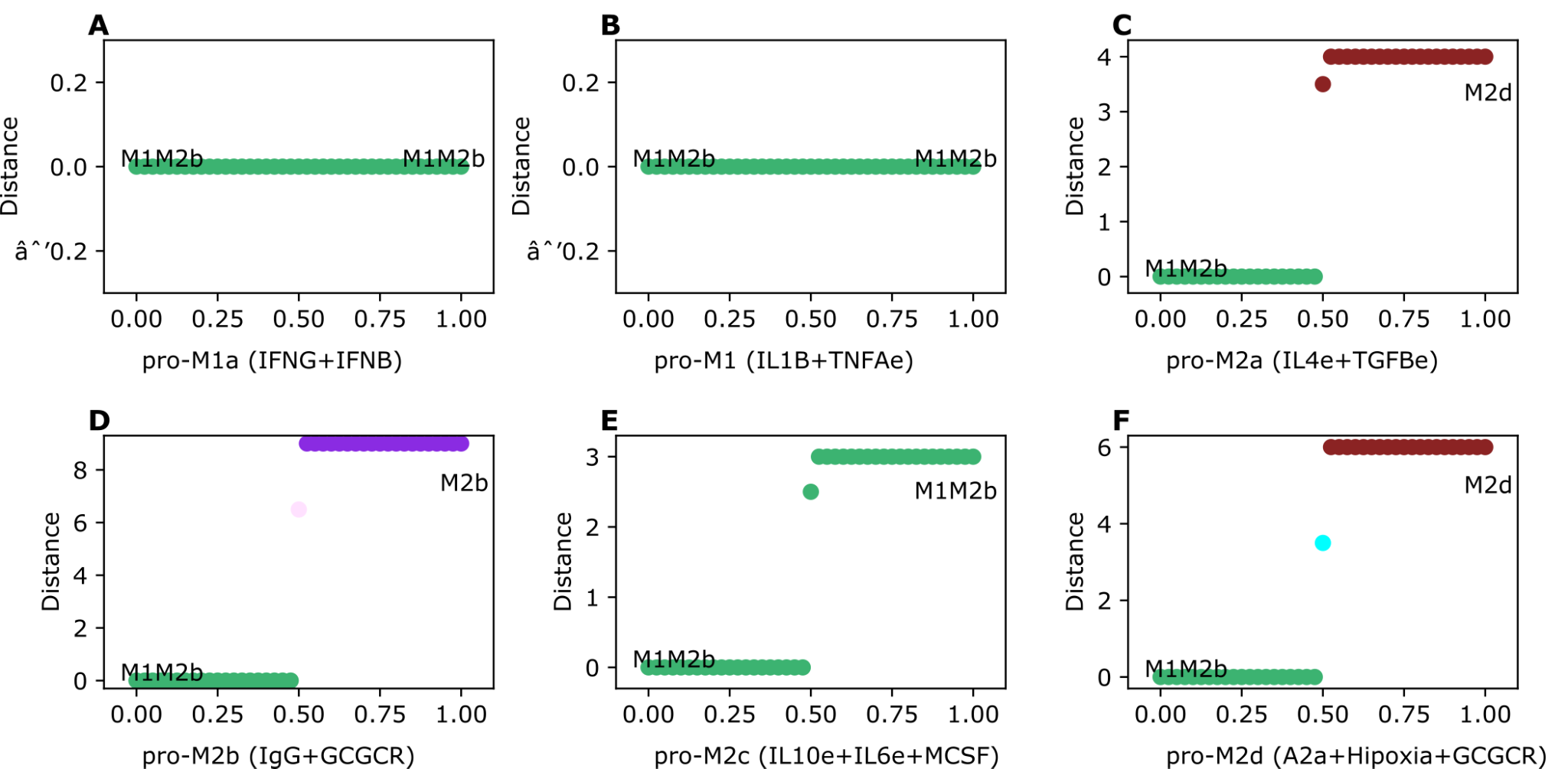


### Figure S2. Macrophage Polarization in different phenotype-associated microenvironments. All panels have as an initial state the M1M2b phenotype. The plot shows in the y-axis the difference between the values of the initial state M0 and the final steady-state, meanwhile the x-axis plots the gradual increase of the exogenous cytokines. (A) Gradual augmentation of the interferon-gamma (IFNG) and interferon-beta (IFNB) in the M1a environment, (B) enhancement of tumor necrosis factor alfa (TNFAe) and interleukin 1-beta (IL1B) for the M1 microenvironment, (C) gradual increasing of interleukin-4 and tumor growth factor, a pro-M2a microenvironment, (D) gradual augmentation of immunoglobulin and glucocorticoids, the pro-M2b microenvironment, (E) gradual enhancement of interleukin-10, macrophage colony-stimulating factor and interleukin-6, the pro-M2c environment, and (F) gradual increase of adenosines, hypoxia, and glucocorticoids, the pro-M2d environment.


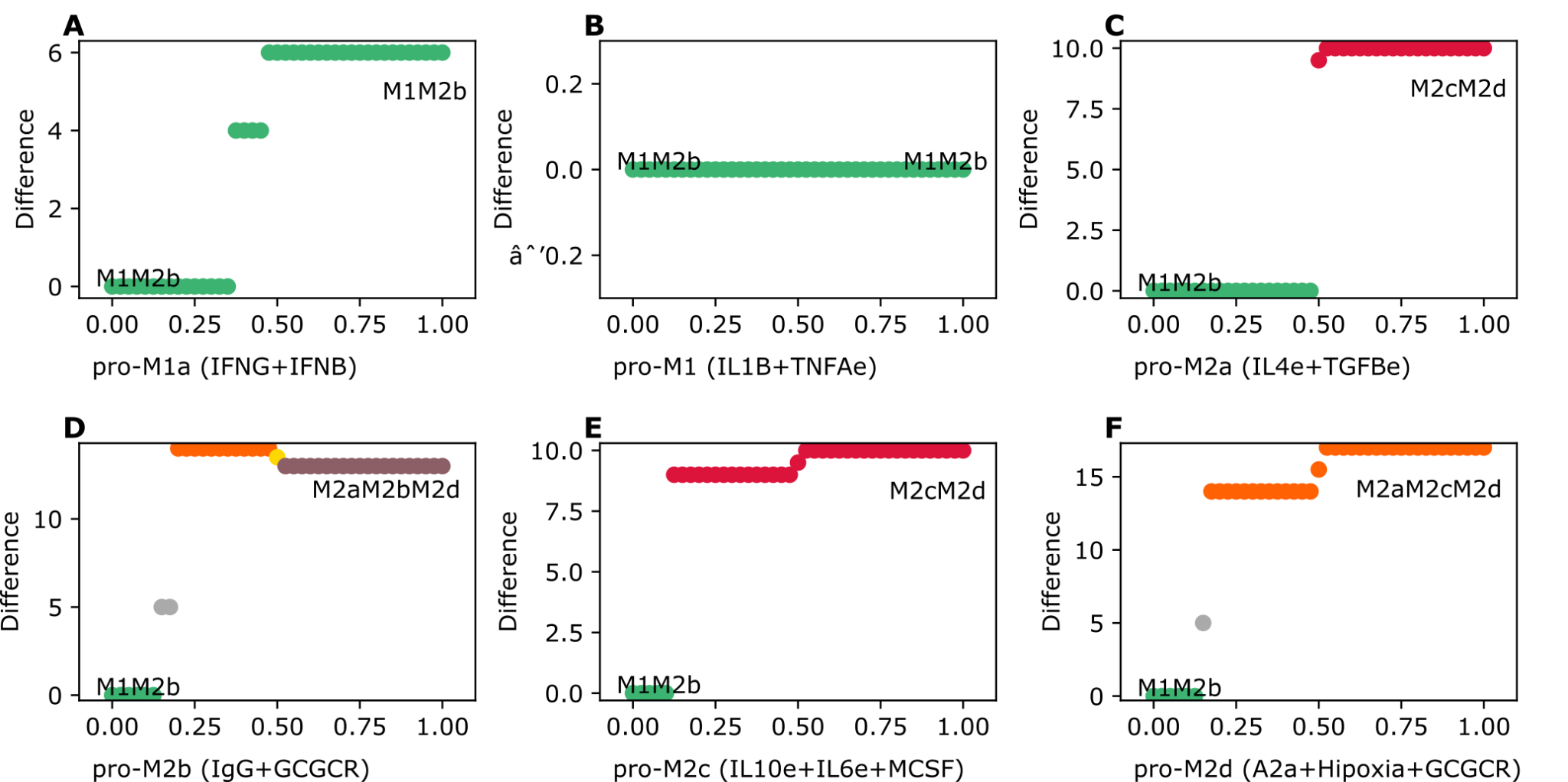


### Figure S3. Macrophage Polarization in different phenotype-associated microenvironments. All panels have as an initial state the M2bM2d phenotype. The plot shows in the y-axis the difference between the values of the initial state M0 and the final steady-state, meanwhile the x-axis plots the gradual increase of the exogenous cytokines. (A) Gradual augmentation of the interferon-gamma (IFNG) and interferon-beta (IFNB) in the M1a environment, (B) enhancement of tumor necrosis factor alfa (TNFAe) and interleukin 1-beta (IL1B) for the M1 microenvironment, (C) gradual increasing of interleukin-4 and tumor growth factor, a pro-M2a microenvironment, (D) gradual augmentation of immunoglobulin and glucocorticoids, the pro-M2b microenvironment, (E) gradual enhancement of interleukin-10, macrophage colony-stimulating factor and interleukin-6, the pro-M2c environment, and (F) gradual increase of adenosines, hypoxia, and glucocorticoids, the pro-M2d environment.


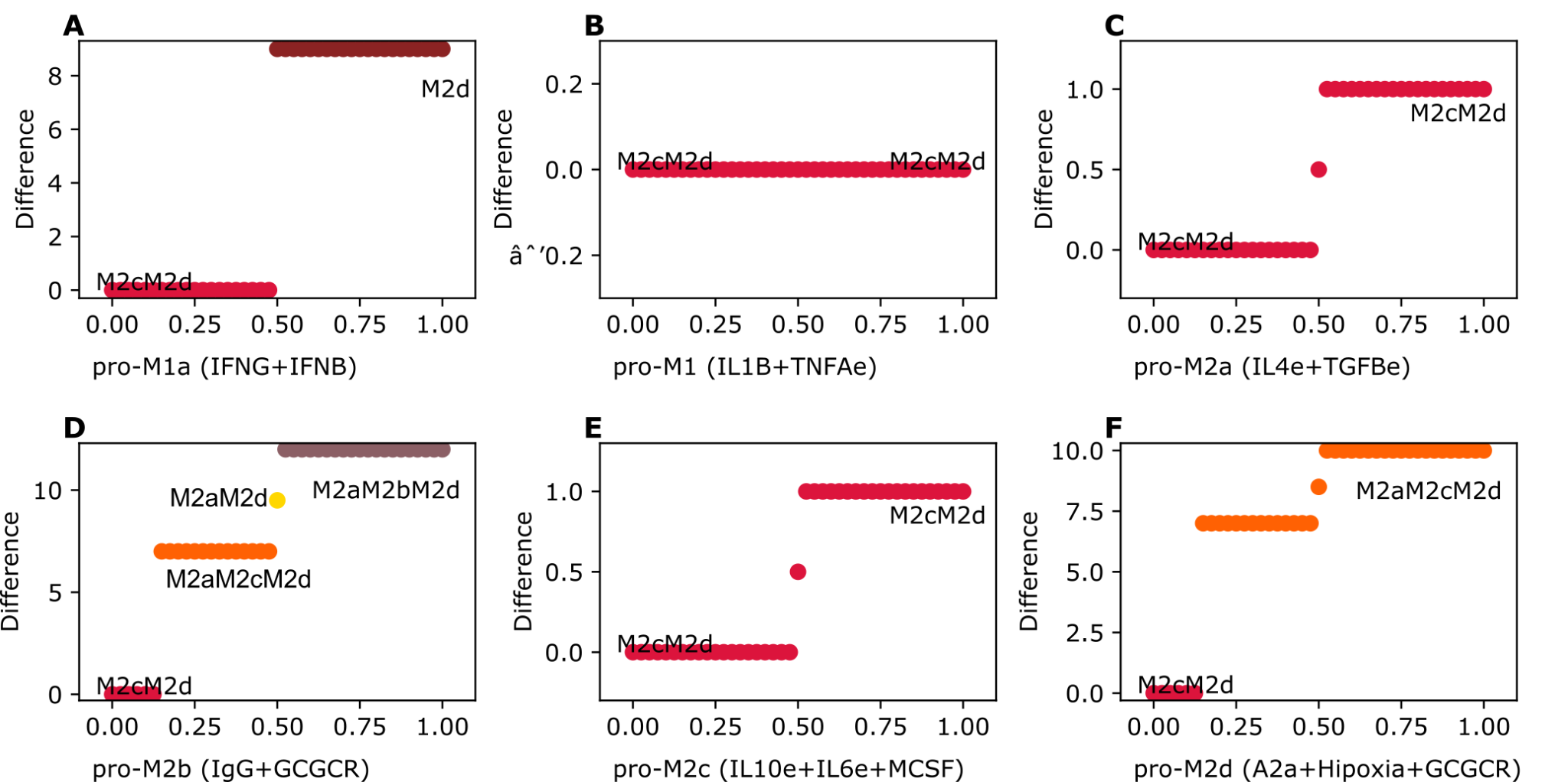


### Figure S4. Macrophage Polarization in different phenotype-associated microenvironments. All panels have as an initial state the M2cM2d phenotype. The plot shows in the y-axis the difference between the values of the initial state M0 and the final steady-state, meanwhile the x-axis plots the gradual increase of the exogenous cytokines. (A) Gradual augmentation of the interferon-gamma (IFNG) and interferon-beta (IFNB) in the M1a environment, (B) enhancement of tumor necrosis factor alfa (TNFAe) and interleukin 1-beta (IL1B) for the M1 microenvironment, (C) gradual increasing of interleukin-4 and tumor growth factor, a pro-M2a microenvironment, (D) gradual augmentation of immunoglobulin and glucocorticoids, the pro-M2b microenvironment, (E) gradual enhancement of interleukin-10, macrophage colony-stimulating factor and interleukin-6, the pro-M2c environment, and (F) gradual increase of adenosines, hypoxia, and glucocorticoids, the pro-M2d environment.


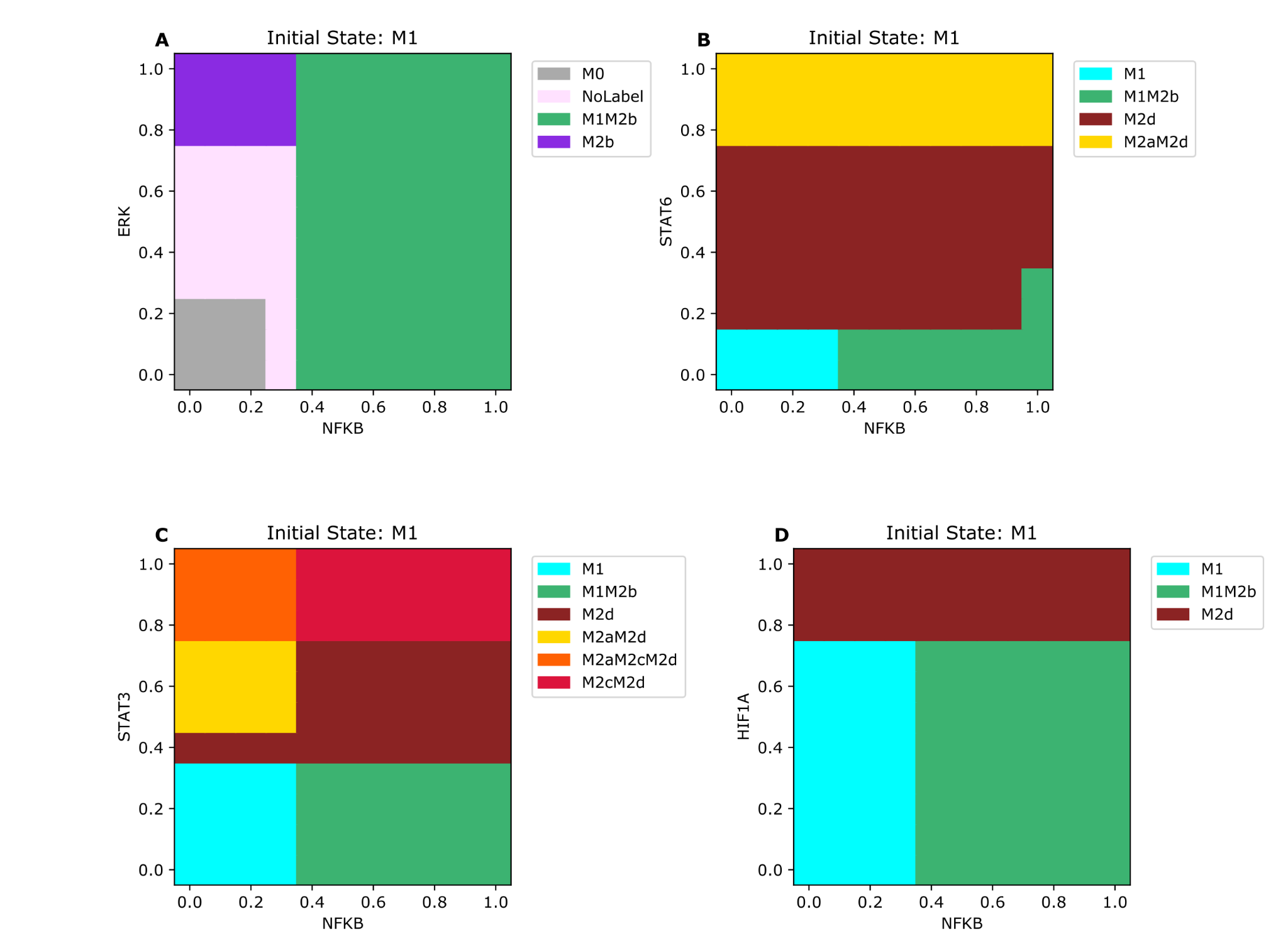


### Figure S5. Phenotype space diagrams for the M1 macrophage phenotype in opposite microenvironments. All panels of the diagram have as an initial state the M1 macrophage (NFкB activated). As well for all panels, NFкB has gradually increased in the opposite macrophage microenvironments (A) Gradual augmentation of ERK transcription factor in a pro-M2b microenvironment, (B) enhancement of STAT6 transcription factor in a pro-M2a microenvironment (C) gradual increasing of the STAT3 transcription factor in a pro-M2c microenvironment, (D) gradual augmentation of HIF1-α in a pro-M2d microenvironment.


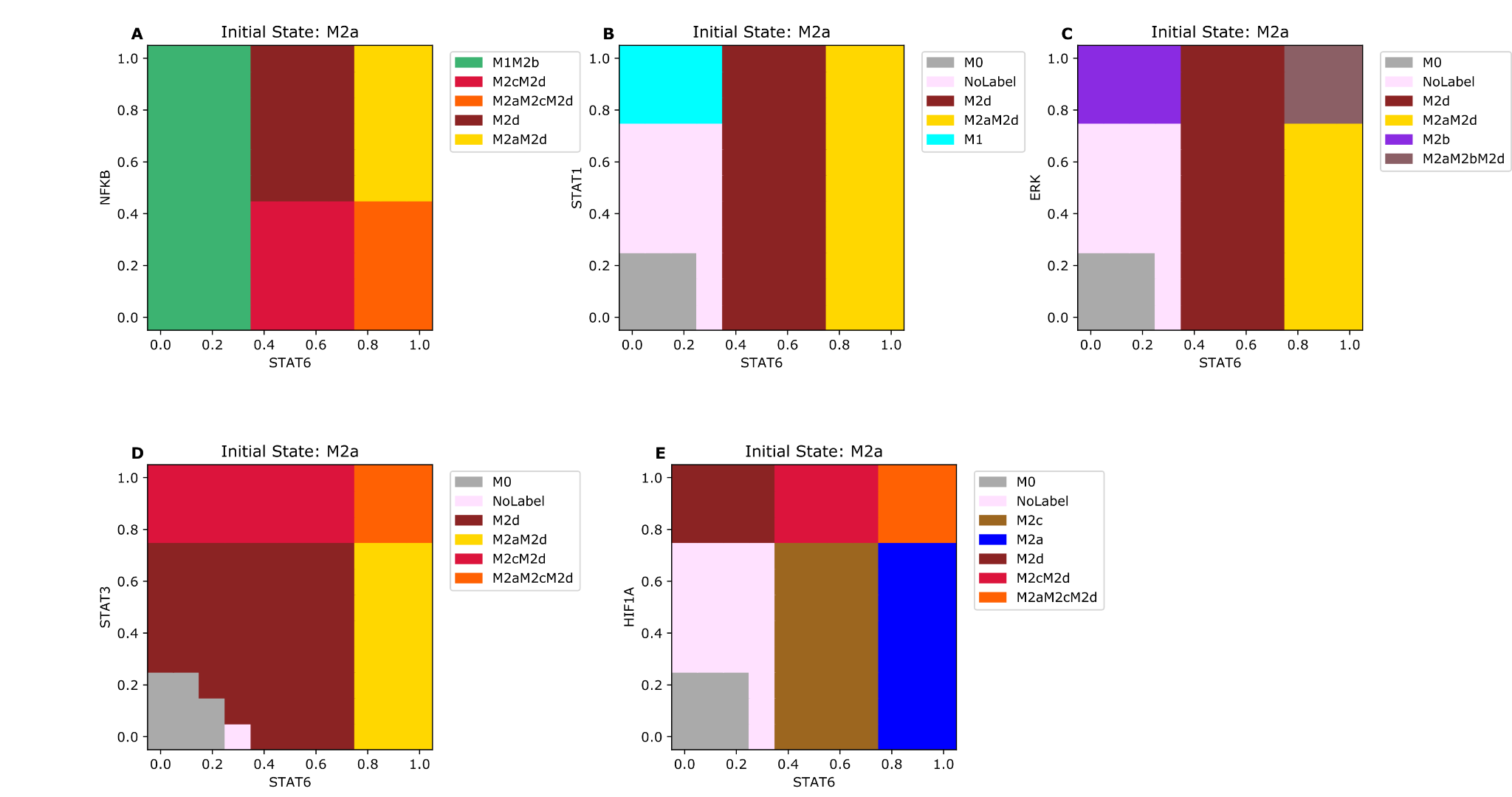


### Figure S6. Phenotype space diagrams for the M2a macrophage phenotype in opposite microenvironments. All panels of the diagram have the M2a macrophage (STAT6 activated) as an initial state. As well for all panels, STAT6 is gradually increased in the opposite macrophage microenvironments (A) Gradual increase of NFкB in a pro-M1 microenvironment, (B) Gradual enhancement of STAT1 in a pro-M1a microenvironment, (C) Gradual augmentation of ERK transcription factor in a pro-M2b microenvironment, (D) gradual enhancement of the STAT3 transcription factor, in a pro-M2c microenvironment, and (E) gradual augmentation of HIF1-α in a pro-M2d microenvironment.


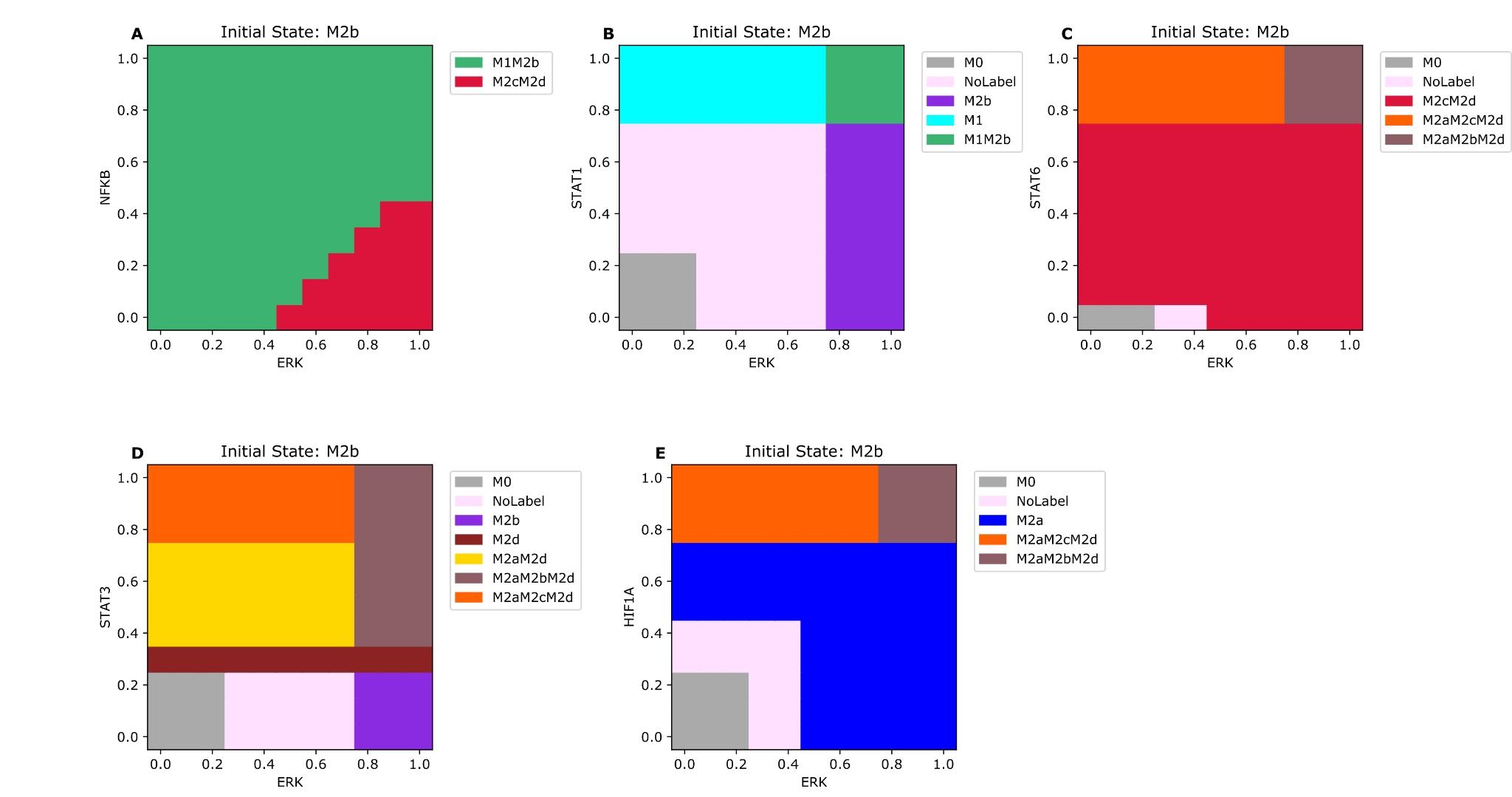


### Figure S7. Phenotype space diagrams for the M2b macrophage phenotype in opposite microenvironments. All diagram panels have the M2b macrophage (ERK activated) as an initial state. As well for all panels, ERK is gradually increased in the opposite macrophage microenvironments (A) Gradual increase of NFкB in a pro-M1 microenvironment, (B) Gradual enhancement of STAT1 in a pro-M1a microenvironment, (C) Gradual augmentation of the STAT6 transcription factor in a pro-M2a microenvironment, (D) gradual enhancement of the STAT3 transcription factor, in a pro-M2c microenvironment, and (E) gradual augmentation of HIF1-α in a pro-M2d microenvironment.


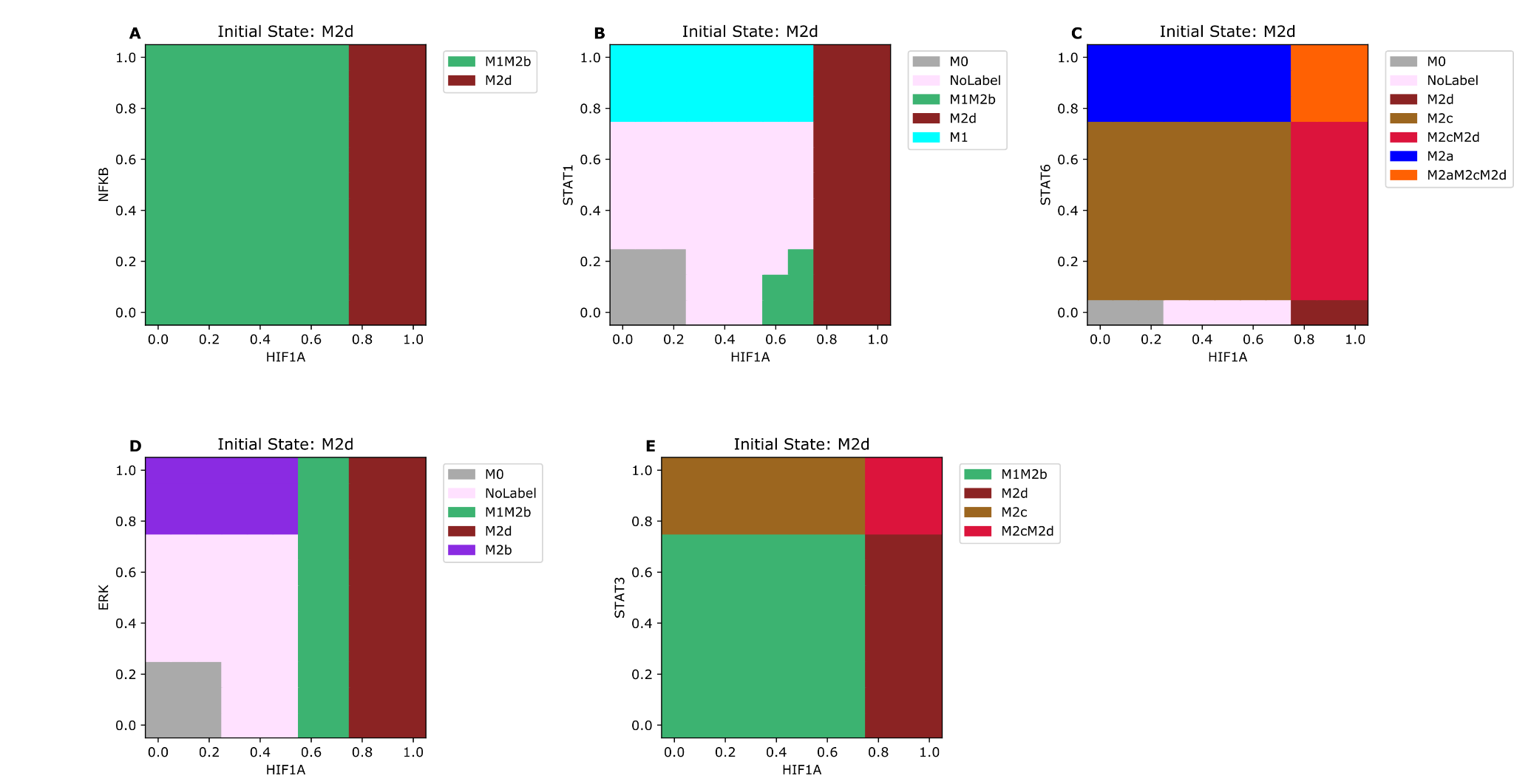


### Figure S8. Phenotype space diagrams for the M2c macrophage phenotype in opposite microenvironments. All diagram panels have the M2c macrophage (HIF1-α activated) as an initial state. As well for all panels, HIF1-α is gradually increased in the opposite macrophage microenvironments (A) Gradual increase of NFкB in a pro-M1 microenvironment, (B) Gradual enhancement of STAT1 in a pro-M1a microenvironment, (C) Gradual augmentation of the STAT6 transcription factor in a pro-M2a microenvironment, (D) gradual increase of the ERK transcription factor in a pro-M2b microenvironment, and (E) gradual enhancement of the STAT3 transcription factor, in a pro-M2c microenvironment.
